# Phased secondary small interfering RNAs in *Camellia sinensis* var. *assamica*

**DOI:** 10.1093/nargab/lqad103

**Published:** 2023-11-24

**Authors:** Angbaji Suo, Jun Yang, Chunyi Mao, Wanran Li, Xingwang Wu, Wenping Xie, Zhengan Yang, Shiyong Guo, Binglian Zheng, Yun Zheng

**Affiliations:** College of Landscape and Horticulture, Yunnan Agricultural University, No. 95 Jinhei Road, 650201 Yunnan, China; School of Criminal Investigation, Yunnan Police College, No. 249 North Jiaochang Road, 650223 Yunnan, China; College of Landscape and Horticulture, Yunnan Agricultural University, No. 95 Jinhei Road, 650201 Yunnan, China; College of Landscape and Horticulture, Yunnan Agricultural University, No. 95 Jinhei Road, 650201 Yunnan, China; College of Landscape and Horticulture, Yunnan Agricultural University, No. 95 Jinhei Road, 650201 Yunnan, China; College of Landscape and Horticulture, Yunnan Agricultural University, No. 95 Jinhei Road, 650201 Yunnan, China; College of Landscape and Horticulture, Yunnan Agricultural University, No. 95 Jinhei Road, 650201 Yunnan, China; College of Landscape and Horticulture, Yunnan Agricultural University, No. 95 Jinhei Road, 650201 Yunnan, China; State Key Laboratory of Genetic Engineering, Ministry of Education Key Laboratory of Biodiversity Sciences and Ecological Engineering, Institute of Plant Biology, School of Life Sciences, Fudan University, No. 220 Handan Road, 200433 Shanghai, China; College of Landscape and Horticulture, Yunnan Agricultural University, No. 95 Jinhei Road, 650201 Yunnan, China

## Abstract

Phased secondary small interfering RNAs (phasiRNAs) in plants play important roles in regulating genome stability, plant development and stress adaption. *Camellia sinensis* var. *assamica* has immense economic, medicinal and cultural significance. However, there are still no studies of phasiRNAs and their putative functions in this valuable plant. We identified 476 and 43 PHAS loci which generated 4290 twenty one nucleotide (nt) and 264 twenty four nt phasiRNAs, respectively. Moreover, the analysis of degradome revealed more than 35000 potential targets for these phasiRNAs. We identified several conserved 21 nt phasiRNA generation pathways in tea plant, including miR390 → TAS3, miR482/miR2118 → NB-LRR, miR393 → F-box, miR828 → MYB/TAS4, and miR7122 → PPR in this study. Furthermore, we found that some transposase and plant mobile domain genes could generate phasiRNAs. Our results show that phasiRNAs target genes in the same family in *cis*- or *trans*-manners, and different members of the same gene family may generate the same phasiRNAs. The phasiRNAs, generated by transposase and plant mobile domain genes, and their targets, suggest that phasiRNAs may be involved in the inhibition of transposable elements in tea plant. To summarize, these results provide a comprehensive view of phasiRNAs in *Camellia sinensis* var. *assamica*.

## Introduction

Small RNAs in plants play important roles in regulating genome stability, plant development and stress adaption (1–3), which can be classified into two main groups: microRNAs (miRNAs) and small interfering RNAs (siRNAs) (4,5). Generally, the lengths of most miRNAs in plant range from 20 to 22 nucleotides (nt), whereas siRNAs are mainly 21 or 24 nt (6,7). Based on the function and biogenesis, siRNAs can be divided into three types: heterochro-matic siRNA (hc-siRNA), natural antisense transcript siRNA (NAT-siRNA) and phased secondary siRNA (phasiRNA) (8–11). When being compared with miRNAs whose precursors are single-strand, siRNAs are produced from long double-stranded RNAs (dsRNAs) (6,12,13).

There are three predominant protein families in biogenesis pathway of phasiRNAs: DICER-LIKE (DCL), ARGONAUTE (AGO) and RNA DEPENDENT RNA polymerase (RDR) proteins (14,15). Firstly, miRNAs with AGO proteins induce site-specific cleavages on the primary precursors of phasiRNAs (PHAS) (16–18). Once triggered by miRNAs, the primary PHAS transcripts are converted into double-stranded RNAs through RDR6. Finally, the resulted dsRNAs are cleaved by DCL4/5 to produce phased fragment RNAs in different lengths (19,20). PhasiRNAs can be encoded from either long non-coding transcripts or coding genes. Several studies also found PHAS loci in the large coding gene families like pentatricopeptide repeat (PPR), F-box auxin receptor, MYB, and nucleotide-binding and leucine-rich repeat (NB-LRR) whose miRNA triggers were miR161/miR7122, miR393, miR828 and miR482/miR2118, respectively (21–27).

PhasiRNAs that repress their target transcripts from other loci of the genome are also called as tasiRNAs, which are originated from non-coding genes (named *TAS*) (8,16,28). *TAS1* and *TAS2* are triggered by miR173, and their tasiRNAs in turn can target mRNAs of PPR proteins (21). *TAS3* derived tasiRNAs, which are triggered by miR390, target the ARF gene families implicated in the developmental transition from juvenile phase to adult phase, leaf morphology, and lateral root growth (16,29). Additionally, miR828 triggers *TAS4* tasiRNAs that target the mRNAs of MYB transcription factors (30).

It has been reported that TAS3-derived tasiRNAs play important roles in leaf development (31) and root development (32,33). Beside TAS3 siRNAs, only a few studies clearly show the functions of phasiRNAs. Guo *et al.* (34) reported that phasiRNAs played a role in seed germination of wheat. Shahid *et al.* (35) reported that a miRNA of parasitic plant *Cuscuta campestris* targeted host messenger RNAs to produce phasiRNAs, which amplified target silencing. Recently, Liang *et al.* (36) showed that an inverted repeat region in Mimulus (monkeyflowers) generated phasiRNAs. One of these phasiRNAs causes change in flower color by targeting a master regulator of floral carotenoid pigmentation (36). More details about the biogenesis and functions of phasiRNAs were reviewed in (11).

Tea, as healthy and nonalcoholic beverage (37), produced from leaves of *Camellia sinensis* (*C. sinensis*), has important economic, medicinal and cultural value (38). Attribute to many of the characteristic secondary metabolites in tea leaves, such as flavonoid, caffeine and theanine, tea has great health and medical benefits to human health (39,40). According to the size of leaf, *C. sinensis* can be divided into two main types: *C. sinensis* var. *assamica* (CSA) with large leaf, and *C. sinensis* var. *sinensis* (CSS) with small leaf (41). Several studies reported miRNAs in *C. sinensis* (42–46), but there are still no reports about phasiRNAs in *C. sinensis*.

Although the biogenesis of 24 nt phasiRNA in plants is poorly studied, their presence in both monocots and eudicots is evident (47–49). Moreover, several studies demonstrated that the 24 nt phasiRNA has a critical role in early seed development (50–52). A research on the function of phasiRNAs in seed development shown that almost 90$\%$ phasiRNAs generated from less than 200 loci, and this situation is conserved in plants (53). Transposable elements (TEs) are widespread in plant genomes. Existing research on the functional relationship of transposons and small RNAs indicated that rice Dicer-like 3 homolog OsDCL3a produces 24 nucleotides siRNAs predominantly associated with miniature inverted repeat transposable elements (49).

In this study, we chose to investigate the phasiRNAs in different organs of CSA (YK-10) by analyzing 9 small RNA libraries with triplicate libraries each from roots, leaves and flowers. We identified 476 twenty one nt PHAS loci and 43 twenty-four nt PHAS loci that produced 4290 phasiRNAs of 21 nt and 264 phasiRNAs of 24 nt, respectively. The conserved miRNA triggers of several phasiRNA biogenesis pathways were also found in this study. The analysis of two degradome sequencing profiles revealed >35 000 putative targets for the phasiRNAs. Our results also revealed that plant mobile domain genes and transposase genes could generate 21 nt phasiRNAs, which subsequently repress genes in these two families, respectively, suggesting that phasiRNA generation from transposon-related elements might represent a novel mechanism to repress the transposons in tea plant, as well as other plants with large genomes. Since TEs were important in the breeding and domestication of plant species (54–57), our results provided potential clues for breeding of tea plant by influencing the TE-derived phasiRNAs or the key protein elements in their generation pathways. These results provide the first picture of phasiRNAs, their biogenesis pathways, and their putative functions in CSA.

## Materials and methods

### The sequencing data sets used

We used nine small RNA-seq libraries from the roots, leaves and flowers of CSA (YK-10). We downloaded the sRNA-seq profiles from the NCBI GEO database with the series accession number, GSE138149. We also uesd two degradomes for one leaf and one root sample of CSA (YK-10), respectively. The length of read is single-end 1×50 bp. The two degradome sequencing profiles were retrieved from the NCBI GEO database with the series accession number GSE138150. Nine RNA-seq libraries were retrieved from the NCBI GEO database with the accession number GSE138148.

### Identification of PHAS loci in *Camellia sinensis* var. *assamica* (YK-10)

The self-development programs to predict PHAS loci and phasiRNAs were reported in previous researches (58–61). Firstly, we removed the unique reads of nine small RNA libraries which can be aligned to The TIGR Plant Repeat Databases (62) and Repbase (r20) (63). Next, the 21 and 24 nt unique sequences were retrieved from all remaining reads. Then, the 21 and 24 nt unique reads were aligned to the assembled genome of CSA (64) by SOAP2 (65), a fast algorithm for aligning a large number of small RNA reads to genomes. We examined the distribution of unique 21 nt and 24 nt sRNAs on genome sequences with a window of 210 nt or 240 nt respectively. Because of double-stranded sRNA with 2-nt overhangs at 3’ end (8,10), the sites of sRNAs on the anti-sense strand were considered in the same phase as sRNAs on the sense strand with a two-nucleotide positive offset. Next, we calculated the *P*-values of the windows by using an early proposed Hypergeometric test (66), and also calculated a phase score for each position of the genome sequence using a method proposed previously in (67).

Furthermore, we extended the window with a *P*-value <0.05 to 100 bp at both 5’- and 3’-ends, and merged the overlapped windows, and kept the smaller *P*-value of overlapped windows as the representing *P*-value of the merged window. The false positive rates of merged windows were tested by using reported method in (68). Eventually, the combined windows with a phase score larger than 5 and multiple test corrected *P*-values of smaller than 0.05 were reported as PHAS loci. The predicted PHAS loci were named by following the rules with its chromosome name and a unique serial number which means the order of PHAS loci on this chromosome. The phased siRNAs, i.e. phasiRNAs, of the predicted PHAS loci were named by adding ‘siR’ and a serial number to the name of the PHAS loci.

### Obtaining putative annotations of the PHAS loci identified

The sequences of the PHAS loci identified were aligned to the several databases with BLASTN (Basic Local Alignment Search Tool for Nucleotide), which is a widely used bioinformatics software tool for nucleotide sequence alignment and similarity searching, to obtain putative annotations of the PHAS loci, including cDNAs of tea plant (64), NCBI nt/nr, RepBase (r20) (63) and The TIGR Plant Repeat Databases (62). The matched genes or RNAs with the smallest E-values were kept as putative annotations of the PHAS loci. Moreover, we used InterPro (69) to identify domains in the PHAS sequences.

### Identification of TAS3 loci

Because the TAS3 derived tasiRNAs, i.e. tasiARFs that target the ARF family genes, are highly conserved (26,59,60,70–72), tasiARFs from *Arabidopsis* and rice were aligned to the genome of tea plant (YK-10) by allowing at most two mismatches. The neighboring sequences of the matched loci were cut out to include 250 nt from both the 5’ and 3’ sides. Then we examined the typical miR390 complementary sites around the conserved tasiRNAs by predicting the miR390 sites on the cut-out sequences with the SeqTar algorithm (73).

### Identification miRNA-triggers of PHAS loci

To identify miRNA triggers of PHAS loci, the sequences of miRNAs of tea plant (46), the predicted PHAS loci (as target cDNAs), and the degradome profiles were input to the SeqTar algorithm (73). These two degradome libraries were also used to identify putative targets of the phasiRNAs. Similarly, the sequences of phasiRNAs, the mRNAs of tea plant (YK-10) (64), and the degradome profiles were input to the SeqTar algorithm (73). We selected the targets with at least one valid read, i.e., read started at the 9th to 11th positions of a miRNA binding site, or targets with less than 4 mismatches for further analysis.

### Identification of deregulated phasiRNAs in different organs

The edgeR program, an R package for identifying deregulated genes in RNA-Seq profiles (74), was used to identify deregulated phasiRNAs in rest different tissues of CSA (YK-10). One flower and one leaf sample (flower2 and leaf41, respectively) were very far from the other samples of the same tissues in the clustering analysis of the expression levels of phasiRNAs (Supplementary Figure S2A). These two samples were then not used to identify deregulated phasiRNAs in different organs. The phasiRNAs with FDR vales smaller than 0.05 were regarded as deregulated ones in different organs.

### Identifying phasiRNA targets in *Camellia sinensis* var. *assamica* (YK-10)

In order to obtain the targets of phasiRNAs, we used the SeqTar pipeline (73) to analyze the sequenced degradome profiles. For targets of phasiRNAs, only those with at least 1 valid read in either the leaf or the root degradome profile and no more than 4 mismatches or those without mismatches were kept in further analysis. Cytoscape (v3.8.0) (75) was used to visualize the PHAS $\longrightarrow$ phasiRNAs ⊣ target networks.

## Results

### Summary of sRNA and degradome sequencing profiles used

To identify PHAS loci and phasiRNAs in tea plant, we used nine small RNA-Seq (sRNA-Seq) profiles from the same RNA samples of CSA (YK-10) (46), a diploid elite cultivar widely grown in southwestern region of China (64). There were at least 20 million sequencing reads for each of these sRNA-seq libraries (Additional file 2: Supplementary Figure S1). After merging reads from all 9 libraries, we finally obtained 195 456 787 qualified reads represented by 78 812 907 unique reads in these 9 sRNA-seq profiles. Approximately 71% of these reads could be mapped to the genome of YK-10 (64), indicating good qualities of these libraries.

Because miRNAs often trigger phasiRNA generation by inducing cleavages of their target transcripts, therefore we also generated two degradome profiles for YK-10, i.e., one leaf and one root, respectively (46). Over 19 million and 16 million reads were obtained from the degradome libraries of leaf and root, respectively.

### Summary of PHAS loci in *Camellia sinensis* var. *assamica* (YK-10)

Nine sRNA sequencing profiles of three tissues, i.e. leaves, roots and flowers, were mapped to reported genome of CSA (64) using SOAP2 (Short Oligonucleotide Alignment Program 2) (65). After that, we predicted PHAS loci and phasiRNAs from the alignment result of SOAP2 by a self-developing program reported previously (58,60,61). Based on a combined criterion of a phase score ≥ 5 and a multiple-test corrected *P* < 0.05, we finally identified 476 and 43 PHAS loci generating 21 and 24 nt phasiRNAs respectively (Additional file 1: Supplementary Table S1 and S2, respectively). These loci encoded 4290 and 264 phasiRNAs of 21 nt and 24 nt, respectively (Additional file 1: Supplementary Table S3 and S4, respectively).

We performed bi-clustering and PCA analysis for normalized reads count of 21 nt phasiRNAs. The results revealed that the samples from the same organs were grouped together (Additional file 2: Supplementary Figure S2A and S2B), suggesting that the expression patterns of phasiRNAs might carry the information of the tissue specific information.

We detected 1332, 1048 and 903 differentially expressed phasiRNAs in comparisons of flower vs leaf, root vs flower, and root vs leaf, respectively (Additional file 1: Supplementary Tables S5–S7, respectively). Compared with flowers, there are 420 phasiRNAs downregulted in leaves, while 912 phasiRNAs were upregulated (Additional file 2: Supplementary Figure S2C). The number of phasiRNAs with high expression level in roots than flowers is 547, and 501 phasiRNAs with low expression level (Additional file 2: Supplementary Figure S2D). In roots and leaves, 340 phasiRNAs presented upregulated trend in roots (Additional file 2: Supplementary Figure S2E).

We aligned the predicted PHAS sequences to NCBI Nucleotide Collection (nr/nt) database, the coding genes of CSA, RepBase and the TIGR Plant Repeat database to obtain putative annotations of the predicted PHAS loci (Additional file 1: Supplementary Table S1 and S2). Both 21 nt and 24 nt PHAS loci were predominantly derived from protein coding genes (Additional file 2: Supplementary Figure S3A and S3B, respectively). And three of the predicted 21 nt PHAS loci were TAS3 loci.

### TAS3 loci in *Camellia sinensis* var. *assamica* (YK-10)

**Figure 1.**
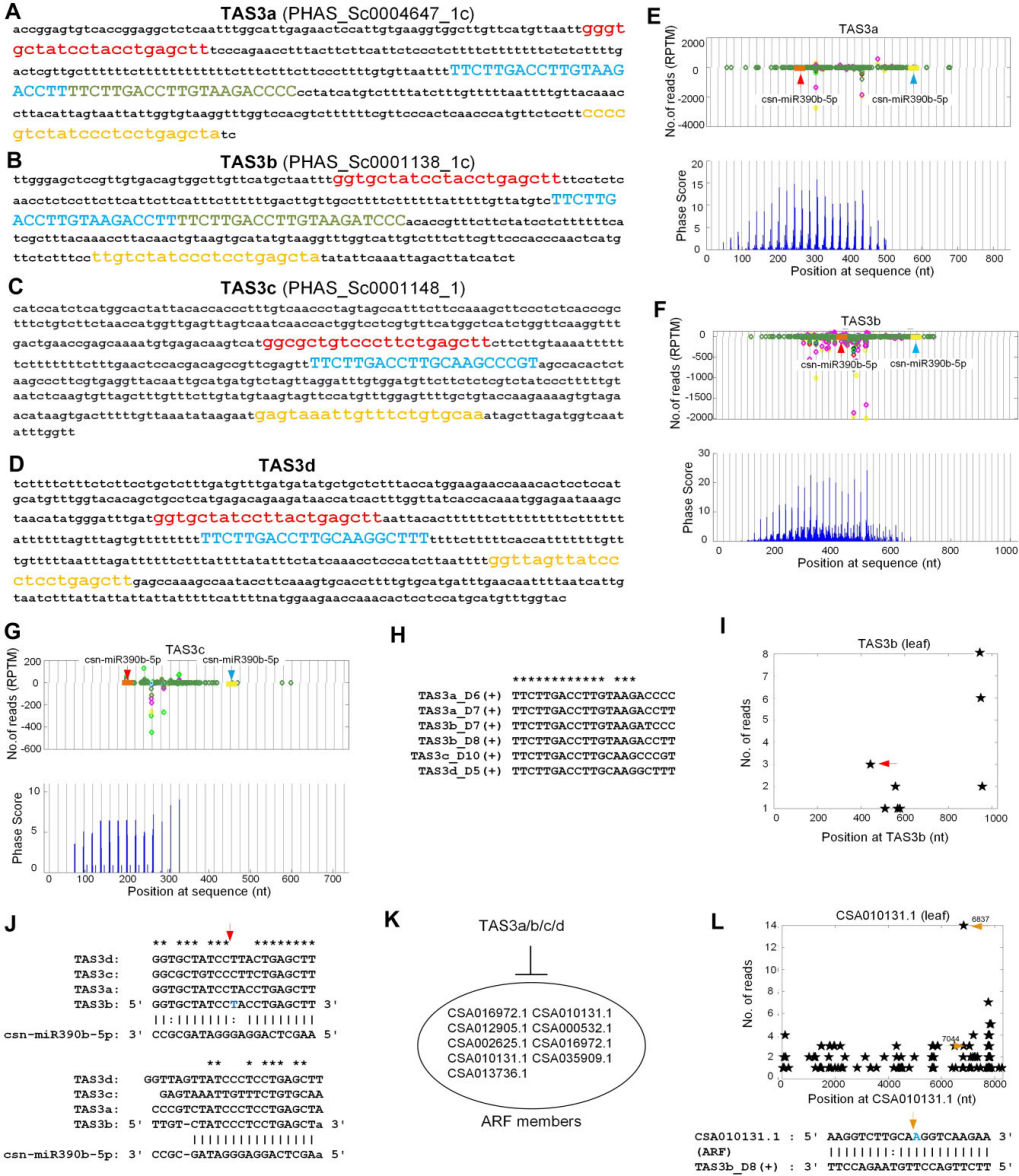
TAS3 loci, tasiRNAs and their targets in *Camellia sinensis* var. *assamica* (YK-10). (**A**) The sequence of TAS3a (PHAS_Sc0004647_1c, i.e., anti-sense strand of PHAS_Sc0004647_1). (**B**) The sequence of TAS3b (PHAS_Sc0001138_1c). (**C**) The sequence of TAS3c (PHAS_Sc0001148_1). (**D**) The sequence of TAS3d. In part (a) and (d) the red and yellow region are the 5’ and 3’ miR390 complementary sites. The blue and green regions in upper cases are the tasiRNAs. (**E**–**G**) The distributions of small RNA reads in the small RNA profiles and Phase Scores on TAS3a, TAS3b and TAS3c, respectively. In Part (E) and (G), the vertical gray lines with distances of 21 nt are the phased positions from the position with the highest phase scores of the PHAS loci. (**H**) The conserved TAS3-derived tasiRNAs in tea plant. (**I**) The T-plot of TAS3b loci, where the red arrow is corresponding to the red arrow in Part (j). (**J**) The 5’ and 3’ miR390 site on TAS3a, TAS3b, TAS3c and TAS3d, respectively. The red arrow indicates the cleavage position on TAS3b induced by miR390 and corresponds to the red arrows in Part (F) and (I). (**K**) The ARF genes which are targeted by tasiRNAs. (**L**) The T-plot and tasiRNA complementary site for one of the ARF genes, CSA010131.1, which is targeted by TAS3b_D8(+).

Three of the identified 21 nt PHAS loci were TAS3 genes as shown in Figure 1A and C. In addition, we found one additional TAS3 gene by aligning conserved tasiRNAs in *Arabidopsis* and rice to genome of tea plant (Additional file 1: Supplementary Table S8). TAS3a was triggered by csn-miR390b-5p at two loci (Figure 1A, E and J) and encoded two conserved tasiARFs, named as TAS3a_D8(+) and TAS3a_D7(+) (the blue and green parts in Figure 1A, respectively), by following the nomenclature of tasiRNAs (16). Similarly, the csn-miR390b-5p was also targeting TAS3b at two complementary sites (Figure 1B, F and J). TAS3b also generated two conserved tasiARFs, i.e., TAS3b_D8(+) and TAS3b_D7(+) (the blue and green parts in Figure 1B, respectively). As noticed in the leaf degradome (Figure 1I), the 5’ miR390 site on TAS3b seemed to be cleavable. Normally, the 3’ miR390 site on TAS3 was cleaved to start the generation of phasiRNAs (16,29). Recently, the 5’ miR390 sites of a few TAS3 loci in Norway spruce (*Picea abies*) were reported to be cleavable (26). TAS3c and TAS3d only encode one tasiARF, respectively (Figure 1C and D, respectively). But these two loci also had two typical miR390 complementary sites around the conserved tasiRNAs (Figure 1C, D and J).

In our sRNA-seq profiles, a lot of phasiRNAs were produced from TAS3a/b/c (Figure 1E–G). In the degradome analysis, we found that the TAS3 derived tasiRNAs (Figure 1H) targeted at least 9 ARF genes in tea plant (Figure 1K). For example, TAS3b_D8(+) targeted CSA010131.1 (one of the ARF genes) as shown in Figure 1L.

### miR482 and miR2118 target NB-LRR transcripts to produce phasiRNAs

As plants are immobile, they have to develop immune system including physical barriers, antimicrobial compounds, pattern recognition receptors and resistance genes to defend the attack of microbial pathogens (76). The majority of disease resistance genes in plants encode NB-LRR proteins (77). Nucleotide-binding and leucine-rich repeat (NB-LRR) proteins that are encoded by plenty of plant R genes interact with pathogen effectors to initiate defense responses (78). NB-ARC domain is a large part of core nucleotide-binding fold in NB-LRR proteins (79).

**Figure 2.**
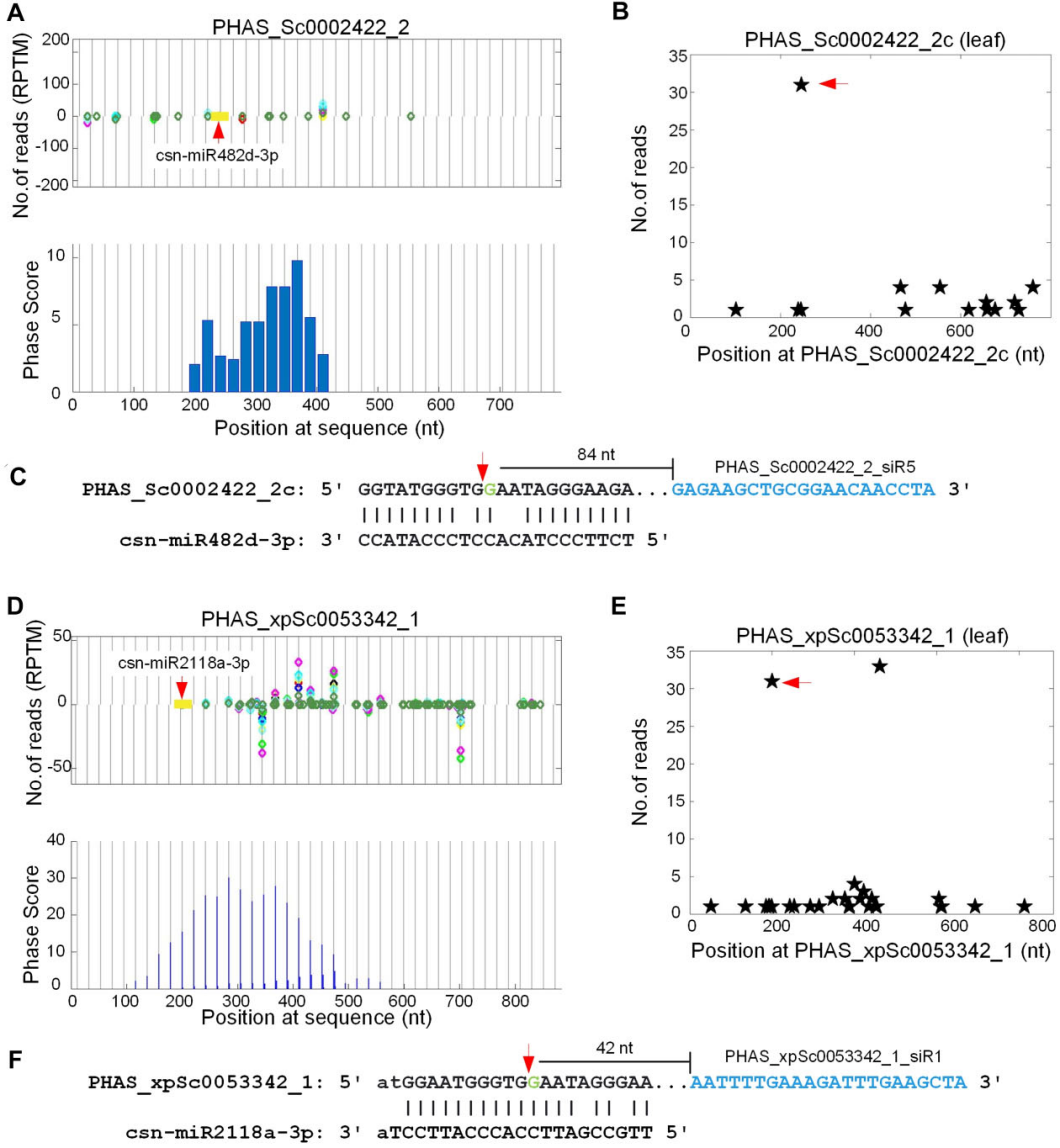
Two 21 nt PHAS loci from NB-ARC genes that are triggered by miR482/miR2118. (**A**) The distribution of 21 nt siRNAs and Phase Scores on PHAS_Sc0000258_1. (**B**, **C**) The T-plot of and complementary site of csn-MIR482e-3p:PHAS_Sc0002422_2, respectively. (**D**) The distribution of 21 nt siRNAs and Phase Scores on PHAS_Sc0000677_1. (**E**, **F**) The T-plot of and complementary site of csn-miR2118a-3p:PHAS_xpSc0053342_1. In Part (A) and (D), the vertical gray lines with distances of 21 nt are the phased positions from the position with the highest phase scores of the PHAS loci. The yellow boxes in the read distribution panel represent the complementary sites of csn-MIR482e-3p and csn-miR2118a-3p, respectively. Sites pointed by miRNAs from above and under zero read line means that miRNAs complement to the plus and minus strand of the predicted PHAS loci, respectively. In Part (C) and (F), the blue regions after the complementary sites are one of the phasiRNAs generated from the PHAS loci.

It was reported that NB-LRR genes as PHAS loci were normally triggered by the miR482/miR2118 family with 22 nt in many plant species (19,22–25,58,60). In this study, we found that twenty-six 21 nt PHAS loci at NB-LRR genes were trigged by the miR482/miR2118 family in tea plant (Additional file 1: Supplementary Table S9). For instance, miR482 and miR2118 triggers two PHAS loci at two NB-LRR genes (PHAS_Sc0002422_2 and PHAS_xpSc0053342_1, respectively) as shown in Figure 2A and D, respectively. After analyzing the leaf degradome profile, it was also verified that miR482/miR2118 made strong cleavages on the two transcripts to initiate the production of phasiRNAs (Figure 2B and E, respectively). PhasiRNAs were generated in the downstream of the miR482 and miR2118 complementary sites (Figure 2C and f, RESPECTIVELY).

In 24 of 26 NB-LRR PHAS loci, the miR482/miR2118 complementary sites located in the NB-ARC domains of these NB-LRR genes (Additional file 2: Supplementary Figure S4A). The two remaining PHAS loci (PHAS_0000232_1 and PHAS_0000232_2 at the bottom of Supplementary Figure S4A might be cleaved by miR482/miR2118 at NB-ARC domains too since their sequences at the miR482/miR2118 complementary sites were the same as those of other NB-LRR PHAS loci. One NB-LRR PHAS loci (PHAS_0001208_1) was triggered by csn-miR482b-5p, which was different from the miR482-3p/miR2118-3p for other loci (Additional file 2: Supplementary Figure S4B). Consequently, the sequence at the csn-miR482b-5p complementary site on PHAS_0001208_1 was different from other NB-LRR PHAS loci (Additional file 2: Supplementary Figure S4C).

### miR828 triggers phasiRNA generation by targeting MYB and TAS4

In plants, MYB transcription factors (TFs) are large gene families, which regulate plant growth and development, physiological activity metabolism, primary and secondary metabolic reactions, and responses to environmental stresses (80,81). In addition to the regulation of key functional genes, the biosynthesis of flavonoids which contributes to the medical value of tea is also regulated by MYB TFs (82).

**Figure 3.**
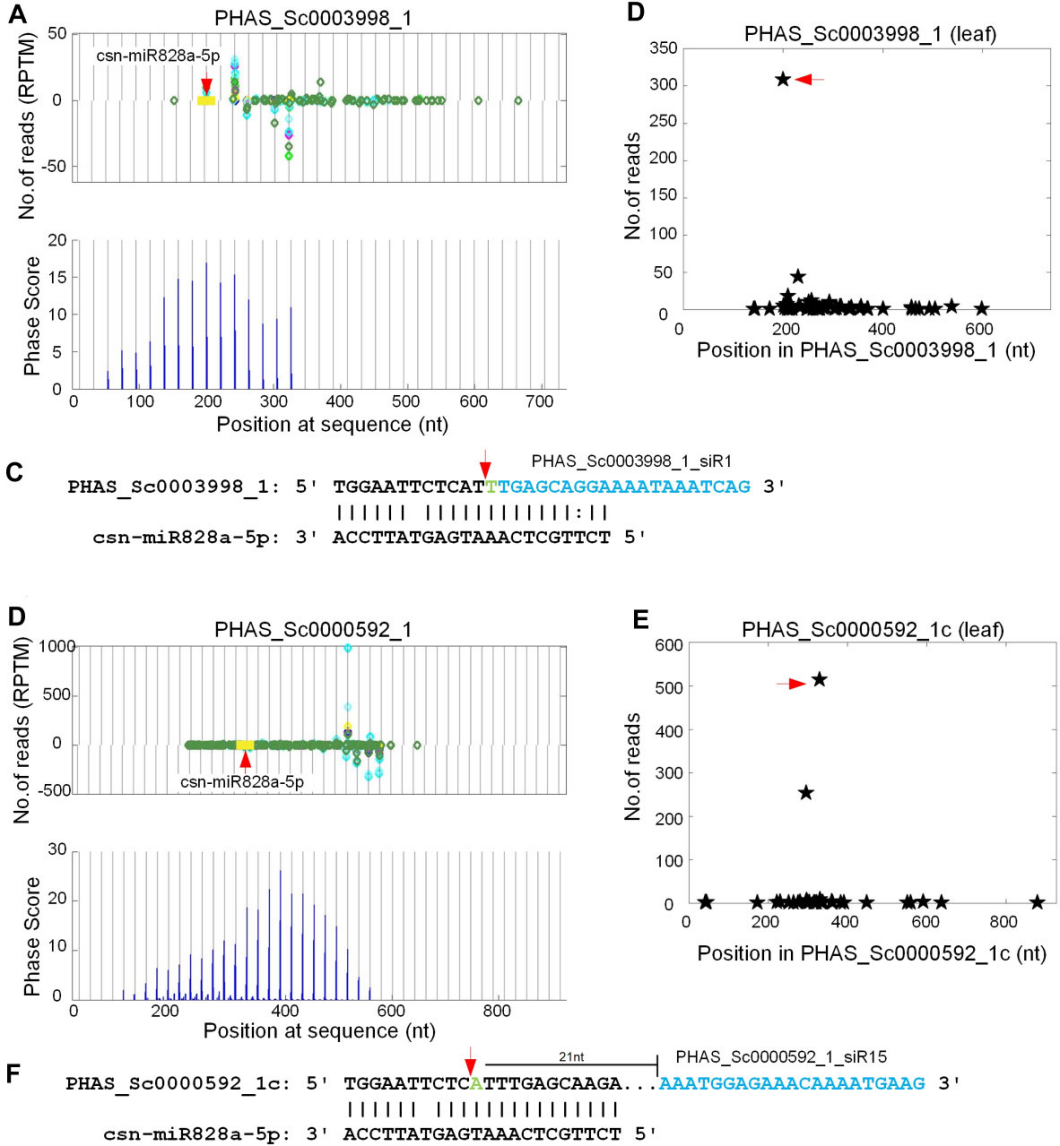
Two PHAS loci at MYB TFs that are triggered by miR828. Legends are similar to those in Figure 2. (**A**) The distribution of 21 nt siRNAs and Phase Scores on PHAS_Sc0003998_1. (**B**, **C**) The T-plot of and complementary sites of csn-miR828a-5p:PHAS_Sc0003998_1, respectively. (**D**) The distribution of 21 nt siRNAs and Phase Scores on PHAS_Sc0000592_1. (**E**, **F**) The T-plot of and complementary sites of csn-miR828a-5p:PHAS_Sc0000592_1, respectively.

In previous studies, it was found that miR828 family targeted MYB TFs to produce phasiRNAs (23,83–85). Our analysis showed that at least 7 PHAS loci triggered by miR828 located at MYB TFs (Additional file 1: Supplementary Table S10 and Additional file 2: Supplementary Figure S5). It was shown in Figure 3A and D that two MYB TFs (PHAS_Sc0003998_1 and PHAS_Sc0000592_1) were targeted by csn-miR828a-5p to induce the generation of phasiRNAs. Meanwhile, as shown in Figure 3 B/C and D/E csn-miR828-5p could induce strong cleavages on these PHAS transcripts in the leaf degradome profile to initiate the generation of phasiRNAs.

Similarly, the reported conserved trigger of TAS4 is miR828 (30). In our analysis, two of PHAS loci were TAS4 genes (Additional file 1: Supplementary Table S11 and Additional file 2: Supplementary Figure S6). As shown in Additional file 2: Supplementary Figure S6A to S6E, after the cleavage sites of miR828, TAS4a as well as TAS4b produced conserved tasiRNA TAS4a_D4(-) and TAS4b_D4(-), respectively. These two TAS4 loci were triggered by csn-miR828a-5p (Additional file 2: Supplementary Figure S6F–G). And the TAS4 derived tasiRNAs targeted at least three MYB genes (Additional file 2: Supplementary Figure S6H), which were reported previously (23,84,85).

### miR7122 targets PPR transcripts to produce phasiRNAs

We detected seven PPR genes initiated by miR7122 to produce phasiRNAs in this study (Additional file 1: Supplementary Table S12). For examples, a lot of phasiRNAs were originated from two loci PHAS_Sc0000048_1 and PHAS_Sc0003224_1, as shown in (Additional file 2: Supplementary Figure S7A and S7B, respectively). These two loci were triggered by csn-miR-7122a-5p (Additional file 2: Supplementary Figure S7B/C and E/F, respectively). A lot of degradome reads were noticed from the complementary sites of csn-miR-7122a-5p(Additional file 2: Supplementary Figure S7B and E, respectively), suggesting the cleavage of PPR transcripts at these sites to produce phasiRNAs. The miR7122 binding sites on these PHAS loci located outside the PPR domains (Additional file 2: Supplementary Figure S8).

Pentatricopeptide repeat (PPR) proteins are one of largest family of sequence-specific-binding proteins in plant (86). They have profound effects in many post-transcriptional processes such as splicing, editing, processing and translation (87). As noticed previously in other species (21,58,88), miR7122, miR1509 and miR173 were originated from a common ancestor and involved in miR7122-TAS-PPR-siRNA pathway in multiple ways (88). Our results suggest that miR7122 directly triggers PPRs to produce phasiRNAs in tea plant, without the involving of TAS or TAS-like genes noticed in other plant species (21,58,88). In *Arabidopsis thaliana*, another miRNA family, miR161 and miR400, also triggered phasiRNA generation by targeting PPRs (21,73). However, we did not found miR161 and PHAS loci triggered by miR161, suggesting that miR161 and PPR loci triggered by miR161 might be non-conserved in tea plant.

### miR393 triggers phasiRNA generation by targeting F-box auxin receptors

**Figure 4.**
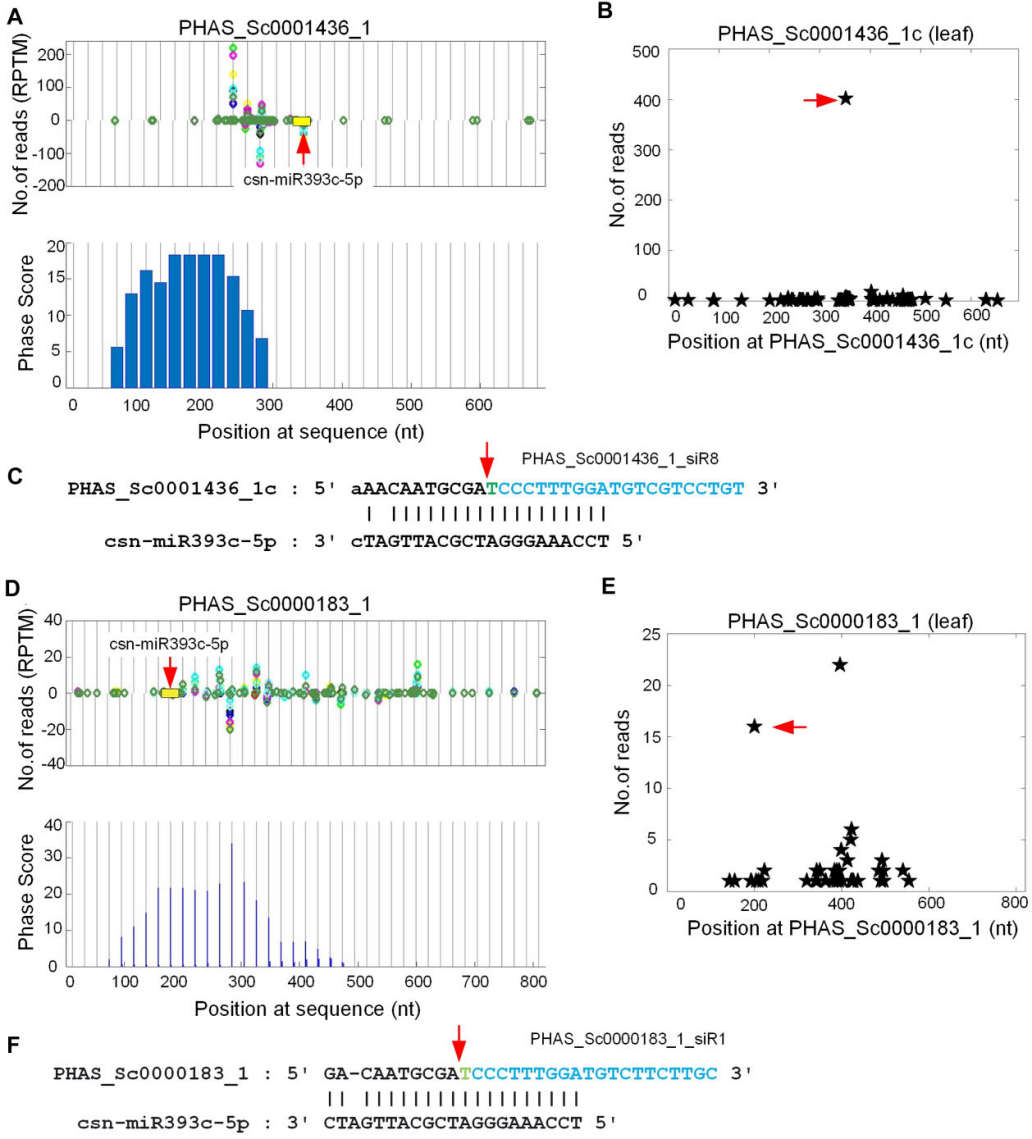
Two PHAS loci at F-Box genes that are triggered by miR393. Legends are similar to those in Figure 2. (**A**) The distribution of 21 nt siRNAs and Phase Scores on PHAS_Sc0001436_1. (**B**, **C**) The T-plot and complementary sites of csn-miR393c-5p:PHAS_Sc0001436_1, respectively. (**D**) The distribution of 21 nt siRNAs and Phase Scores on PHAS_Sc0000183_1. (**E**, **F**) The T-plot and complementary sites of csn-miR393c-5p:PHAS_Sc0000183_1, respectively.

We found that miR393c triggers seven PHAS loci from TIR1 (transport inhibitor response 1)/AFB (auxin signalling F-box) genes (Additional file 1: Supplementary Table S13). For example, csn-miR393c-5p targeted PHAS_Sc0001436_1c and PHAS_Sc0000183_1, F-box like genes, at complementary sites with clear cleavage signals in our degradome libraries (Figure 4A to 4F).

It is known that the miR393 family targets F-box auxin receptors, which is a highly conserved miRNA:target relation (21,73,89–93). miR393 could trigger generation of phasiRNAs by targeting F-box genes in different plant species, such as *Arabidopsis* (27), Norway spruce (26) and *Populus trichocarpa* (94). Our results suggest that miR393 also triggers phasiRNA generation by targeting F-box transcripts in tea plant too.

### PHAS loci at plant mobile domain and transposase genes

**Figure 5.**
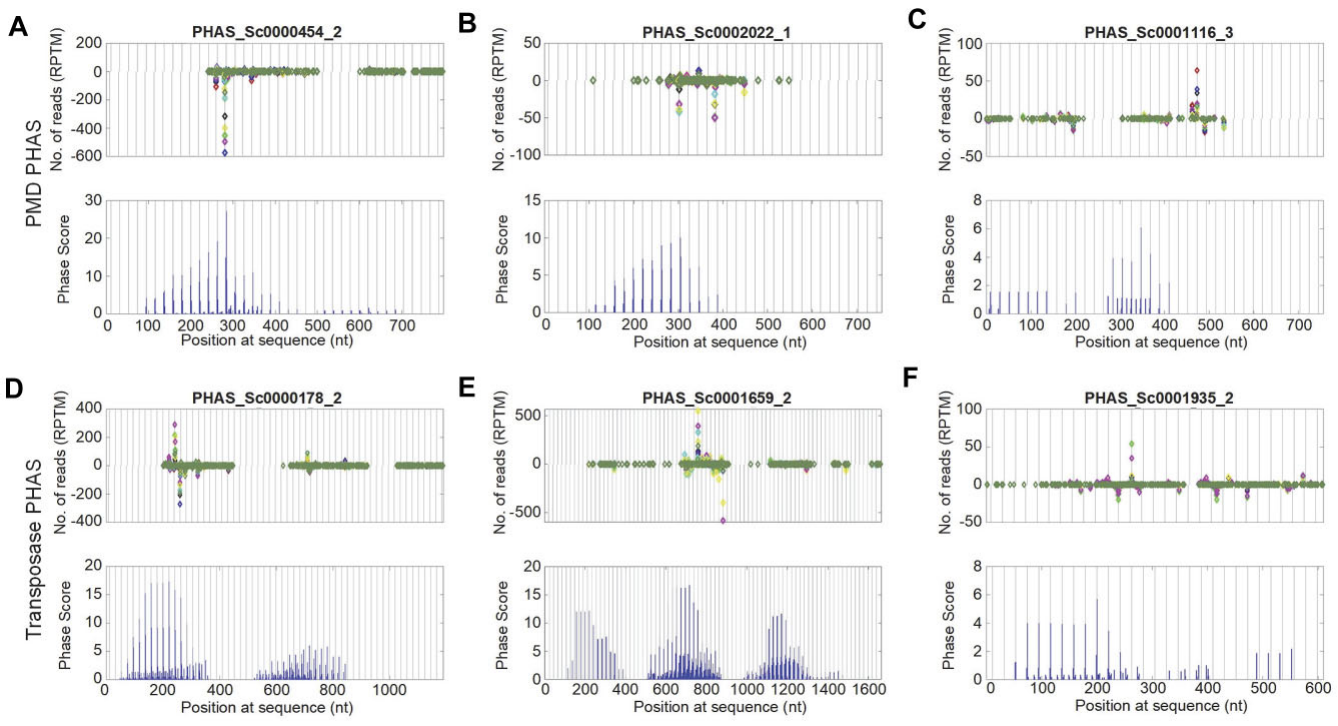
Selected PHAS loci at plant mobile domain and plant transposase genes. Legends are similar to those in Figure 2. (**A**–**C**) The distributions of 21 nt siRNAs and Phase Scores on three PHAS loci at plant mobile domain (PMD) genes, i.e. PHAS_Sc0000545_2, PHAS_Sc0002022_1 and PHAS_Sc0001116_3, respectively. (**D**–**F**) The distributions of 21 nt siRNAs and Phase Scores on three PHAS loci at plant transposase genes, i.e. PHAS_Sc0000178_2, PHAS_Sc0001659_2 and PHAS_Sc00001935_2, respectively.

Plant mobile domain (PMD) proteins are derived from transposable elements (TEs) and play important roles in TE silencing, maintaining genome stability, and regulation of developmental processes (95). We found 175 PHAS loci at plant mobile domain genes (Figure 5A–C, and Additional file 1: Supplementary Table S14). Our results indicated that most PMD genes have very limited expression in roots, leaves, and flowers of tea plant (Additional file 1: Supplementary Table S15 and Additional file 2: Supplementary Figure S9A).

We identified 8 PHAS loci from 4 different transposase genes (Ptta/En/Spm family) in tea plant (Figure 5D to 5F, and Additional file 1: Supplementary Table S16). Similar to PMD genes, the express levels of the transposase genes were also very limited (Additional file 1: Supplementary Table S17 and Additional file 2: Supplementary figure S9B).

### PhasiRNA targets in *Camellia sinensis* var. *assamica* (YK-10)

We investigated the potential targets of the identified phasiRNAs. Based on the targets with at least 1 valid degradome read in either the leaf or root degradome and less than 4 mismatches, we finally identified 35441 and 139 targets of 21 and 24 nt phasiRNAs, respectively (Additional file 1: Supplementary Table S18 and S19, respectively). Among targets of 21 nt phasiRNAs, 29 were *cis*-targets, i.e. the phasiRNAs targets the transcripts from their generation PHAS loci.

**Figure 6.**
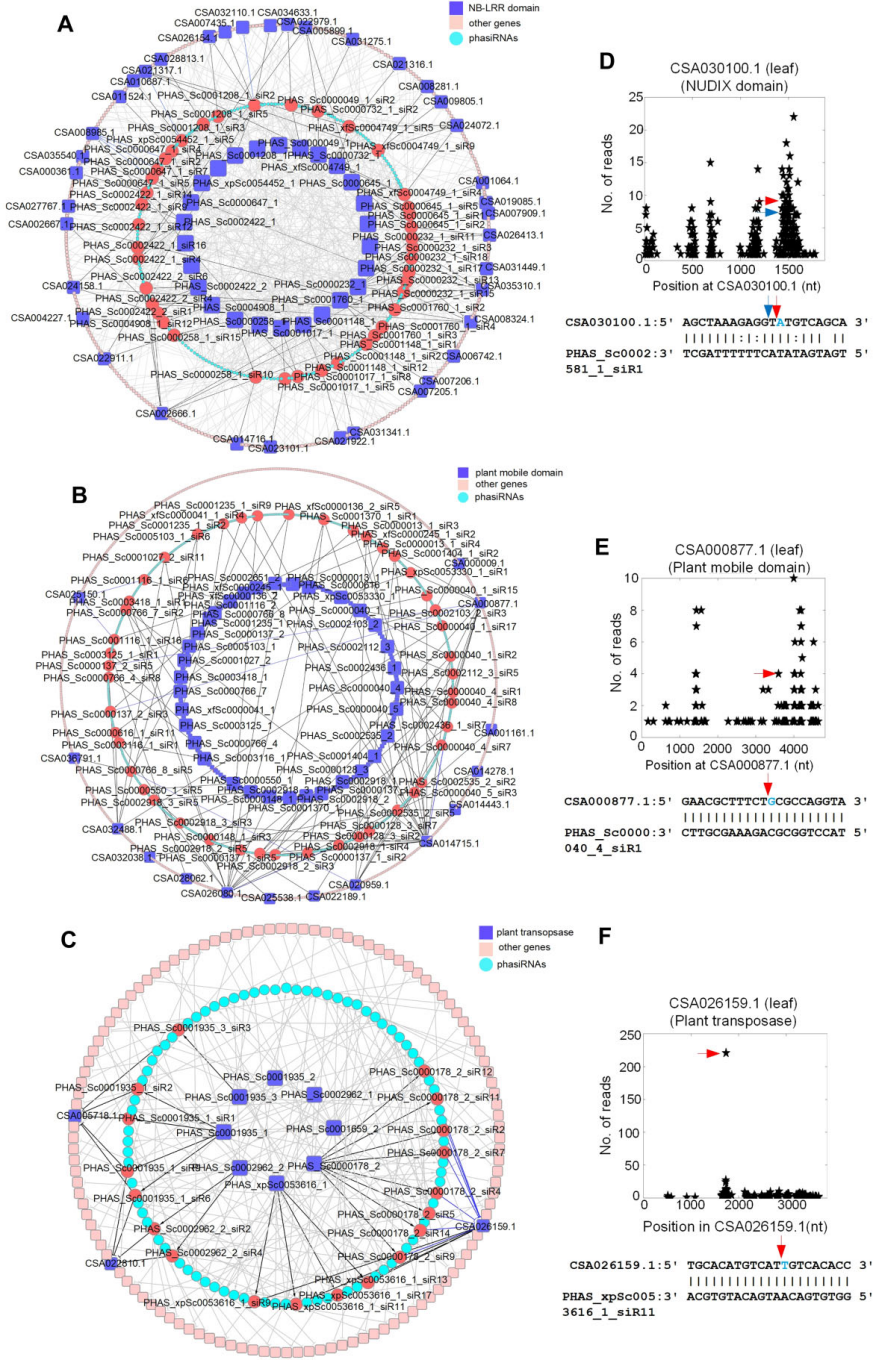
The regulatory networks of phasiRNAs derived from NB-LRR, plant mobile domain and plant transposase genes. (**A**) The regulatory network of phasiRNAs generated from NB-LRR genes. The squares in the central circle are the NB-LRR genes that generate the phasiRNAs in the middle circle. The 40 red circles are phasiRNAs that target at least one NB-LRR genes in either *cis* or *trans*. The squares in the outer circle are the putative targets. The sharp arrow from a gene to a phasiRNA means that the phasiRNA is produced from the PHAS loci at the gene. The blunt arrow from a phasiRNA to a gene means that the phasiRNA directly targets the gene based on the degradome analysis. Blue, black and grey arrows indicate that *cis*-targets, genes in the same family as the PHAS loci, and other genes, respectively. For legibility, only 172 of the 210 phasiRNAs generated from the NB-LRR genes in Additional file 1: Supplementary Table S21 were included in this figure. (**B**) The regulatory network of phasiRNAs generated from plant mobile domain genes. Legend are similar to those in Part (A). (**C**) The regulatory network of phasiRNAs generated from plant transopsase genes. Legend are similar to those in Part (A). (**D**) The T-plot of CSA030100.1 targeted by PHAS_Sc0002581_1_siR1, generated by an NB-LRR gene. (**E**) The T-plot of a plant mobile domain gene (CSA000877.1) targeted by PHAS_Sc0000040_4_siR1, generated by another plant mobile domain gene. (**F**) The T-plot of a plant transposase gene (CSA026159.1) targeted by PHAS_xpSc0053636_1_siR11, generated by another plant transopsase gene.

It was interesting that 519 phasiRNAs were generated from more than one PHAS loci (Additional file 1: Supplementary Table S20). As shown in Figure 6A, 26 NB-LRR genes generated at least 61 different phasiRNAs originated from more than one PHAS loci (Additional file 1: Supplementary Table S21). These phasiRNAs subsequently targeted their generation loci (i.e., *cis*-targets), other NB-LRR genes and other genes (Figure 6A and Additional file 1: Supplementary Table S22). One of the *trans*-targets of phasiRNAs generated from NB-LRR genes were shown in Figure 6D. Similarly, in Figure 6B, 17 plant mobile domain genes generated more than 1000 phasiRNAs, which subsequently targeted their generation loci, other plant mobile domain genes and other genes (Additional file 1: Supplementary Tables S23 and S24). One of the *cis*-targets, i.e. CSA000877.1 was shown in Figure 6E.

Although the plant transopsase genes did not produce as many phasiRNAs as NB-LRR or plant mobile domain genes did, it also followed similar pattern that one PHAS loci generated more than one phasiRNAs (Figure 6C and Additional file 1: Supplementary Table S25). And these phasiRNAs also targeted their generation loci, other plant transposase genes, or other genes (Additional file 1: Supplementary Table S26). For example, one of the phasiRNAs derived from transposase genes targeted another transposase gene (CSA026159.1) in-*trans*, as shown in Figure 6F. The phasiRNA induced repression may contribute to the limited expression of most PMD and plant transposase genes (as shown in Additional file 2: Supplementary Figure S9).

Moreover, it was also noticed that phasiRNAs derived from PPR, MYB and F-Box gene families (Additional file 1: Supplementary Tables S27, S29 and S31, respectively) also targeted their own generation loci, genes of the same family, and other genes (Additional file 2: Supplementary Figure S10 and Additional file 1: Supplementary Tables S28, S30 and S32, respectively). For examples, several phasiRNAs targeted other gene families in both leaf and root degradome profiles (Additional file 2: Supplementary Figure S11).

In summary, these results suggest that phasiRNAs involved in the self-regulation of their generation loci or the genes of the same families. And some phasiRNAs may targeted genes in other families as well.

### Functional analysis of target genes of deregulated phasiRNAs

We applied to KOBAS (v3.0) to analyze the enriched GO terms and KEGG pathways of target genes of deregulated phasiRNAs in different organs. As shown in additional file 2: Supplementary Figures S12–S14, in all three tissues, majority of functions and pathways in target genes of deregulated phasiRNAs were similar. But some unique GO terms or pathways were also noticed. Compared with leaves, malate dehydrogenase activity had higher enrichment level in flowers (Additional file 2: Supplementary Figure S12A). In addition, starch and sucrose metabolic as well as metabolic pathways were also more enriched in flowers than in leaves (Additional file 2: Supplementary Figure S12C). The function of target genes of both downregulated and upregulated phasiRNAs had relatively little difference in roots and leaves, except that embryo sac development was enriched in targets of downregulated phasiRNAs in roots and malic enzyme activity was enriched in targets of upregulated phasiRNAs in roots (Additional file 2: Supplementary Figure S13A, B). The GO term of Metabolic pathways was enriched in targets of downregulated phasiRNAs in roots compared to flowers (Additional file 2: Supplementary Figure S14C).

## Discussion

Tea is good for human health and has immense economic, medicinal as well as cultural significance. However, there are still no researches about phasiRNAs and their functions in tea plant. Therefore, we analyzed 9 small RNA libraries from three different organs of CSA (YK-10). After carefully analyzing these sRNA sequencing profiles, we identified 476 and 43 PHAS loci, encoding 4290 twenty one nt and 264 twenty four nt phasiRNAs, respectively.

In tea plant, miR390 targeted at least four TAS3 loci to generate tasiARFs. miR482/miR2118 family induced phasiRNAs by targeting NB-ARC domains in NB-LRR genes. miR828 triggered generation of phasiRNAs by targeting MYB domains in MYB transcription factors, which played important roles in the biosynthesis of flavonoids in tea plant. miR828 also induced tasiRNA generation by targeting at least two TAS4 loci in tea plant. The TAS4 derived tasiRNAs subsequently repressed MYB genes, therefore forming a miR828-TAS4-MYB regulatory pathway. Finally, miR7112 triggers PPR transcripts to produce phasiRNAs and miR393 could trigger phasiRNA generation by targeting F-box auxin receptors in tea plant.

As reported previously (21–23,58,73,83–85,88,96), our results supported that phasiRNAs generated from NB-LRR, MYB, F-box, and PPR genes could target genes of the same families in tea plant. Furthermore, our results suggested that phasiRNAs were generated from two TE related gene families, i.e. PMD and plant transposase genes.

TE-derived siRNAs were noticed in other plants (97–102). In Arabidopsis, it was postulated that epigenetically activated small interfering RNAs (easiRNAs) derived from TEs were produced to protect the genome from TE-mediated epigenomic instability, by targeting some TE related genes, such as *ATHILA ORF1*, *ATCOPIA* integrase, and *CACTA* transposase (99). PMD family proteins are derived from TEs (95,103). Two PMD proteins, MAIL1 and MAIN, defined an alternative silencing pathway independent of DNA methylation and short interfering RNAs (103). Recently, it was reported that MAIN and MAIL1 interacted with phosphoprotein phosphatase (PPP) protein PP7-like (PP7L) and involved in the silencing of TEs in *Arabidopsis* (95). Our results indicated that PMD transcripts could generate 21 nt phasiRNAs which were subsequently employed to repress PMD transcripts in *cis* and *trans*, in similar ways to phasiRNA generation pathways noticed in NB-LRR and PPR genes. Similarly, we also found that transposase genes, also encoded by TEs, in tea plant could generate phasiRNAs. Transposase is an enzyme that binds to the end of a transposon and catalyses its movement to new loci in the genome by ‘cut-and-paste’ or ‘copy-and-paste’ mechanism (104). Transposases are the most abundant and most ubiquitous genes in nature (104). An active DNA transposase expresses in majority of childhood solid tumors and causes DNA rearrangements to drive tumor development (105). Due to its detrimental to the stability of genome, the activities of transposases were inhibited (106). Our results suggest that some transposase genes could generate phasiRNAs in tea plant, which subsequently repressed transposase genes.

The genome of tea plant is of approximately 3 Gb (64,107), which is much larger than those of other sequenced plant genomes, such as maize (2.3 Gb) (108), lotus (929 Mb) (109), pineapple (526 Mb) (110), rice (466 Mb) (111) and Arabidopsis (120 Mb) (112). The amplification of long terminal repeat retrotransposons, representing approximately 2/3 of the whole genome, is the main reason of the expansion of genome size of tea plant (64,107). Therefore, TEs in tea plant genome must be repressed effectively, probably in a manner previously unnoticed in other plant species. Our results suggest that phasiRNAs derived from TE elements, such as PMD genes and transposase genes, might represent a novel mechanism to silence TEs in plants, especially, those with high contents of TEs in their genomes.

A few TE-derived siRNAs had been demonstrated to have *trans*-targets (97,98,100). For examples, when TEs are globally epigenetically activated and transcribed by Pol II, siRNA854 is derived in its 21–22 nt form from the Arabidopsis *Athila6A* family of centromeric gypsy LTR retrotransposons and inhibits translation of UBP1b (97). And several other TE-derived siRNAs target genic mRNAs in *trans*, such as AMS and HHP2 in Arabidopsis (98). Paternally expressed gene PEG2 sequesters siRNA854 in endosperm, and overexpression of PEG2 depletes siRNA854 and establishes a reproductive barrier between plants of different chromosome numbers (101). Another group also reported that depletion of paternal easiRNAs bypasses the triploid block in Arabidopsis (102). In rice, a TE siRNA, TE-siR815, generated by WRKY45-1, represses ST1 which results in pathogen susceptibility (100). Our results demonstrate that PMD and plant transposase genes produce 21 nt phasiRNAs in tea plant, which may involve in repression of their homologous TE-gene families, respectively (Figure 6B and C, respectively). Since the PMD and plant transposase derived phasiRNAs also target many other genes, they may have other biological functions to be explored in the future.

In *DECREASED DNA METHYLATION 1 (ddm1)* mutant of Arabidopsis, more than 50 miRNAs target thousands of transposon transcripts and trigger generation of 21 nt easiRNAs with the processing of RDR6 (99). Our results suggested that several PHAS at PMD and plant transposase genes might be triggered by several miRNAs, i.e. miR1318-5p, miR172m-5p, miR1863b-5p, miR2673a-5p, miR399d-5p, miR408-3p, miR7782m-5p for PMD genes and miR535c1-3p, miR7782c-3p, miR171r-5p for plant transposase genes respectively (Supplementary Tables S14 and S16, respectively). In a study of Arabidopsis (99), degradome reads are detected at the miRNA complementary sites on transposon transcripts in *ddm1* only, and not found in Col-0 (wild type). There were no degradome reads at the miRNA complementary sites on the PMD and plant transposase transcripts in our degradome profiles of leaf and root samples of tea plant. Therefore, our result is consistent with the latent miRNA-directed easiRNA mechanism which is only activated only when TEs are epigenetically reactivated during reprogramming of germ line (99).

TEs play important roles in domestication and/or breeding of plant species (54–57). The phasiRNAs derived from TE elements, such as PMD and plant transposase genes, in tea plant suggest that it might be a feasible approach to manipulate the TE-derived phasiRNAs or key protein factors (such as RDR6 and DCL4) in phasiRNA generation pathways to accelerate and improve the breeding of tea plant.

In summary, our results provide novel insights into the TE-derived siRNAs in plants.

## Supplementary Material

lqad103_Supplemental_Files

## Data Availability

The small RNA and degradome sequencing profiles were available in the NCBI GEO database under the series accession number GSE138151.
