# Supplementary material for: Phased secondary small interfering RNAs in Camellia sinensis var. assamica
Source: NAR Genom Bioinform. 2023 Nov 24;5(4):lqad103. doi: 10.1093/nargab/lqad103 (PMC10673657; doi:10.1093/nargab/lqad103)
Supplement: lqad103_Supplemental_Files [file lqad103_supplemental_files.zip › Additional file2-v14.docx]

**
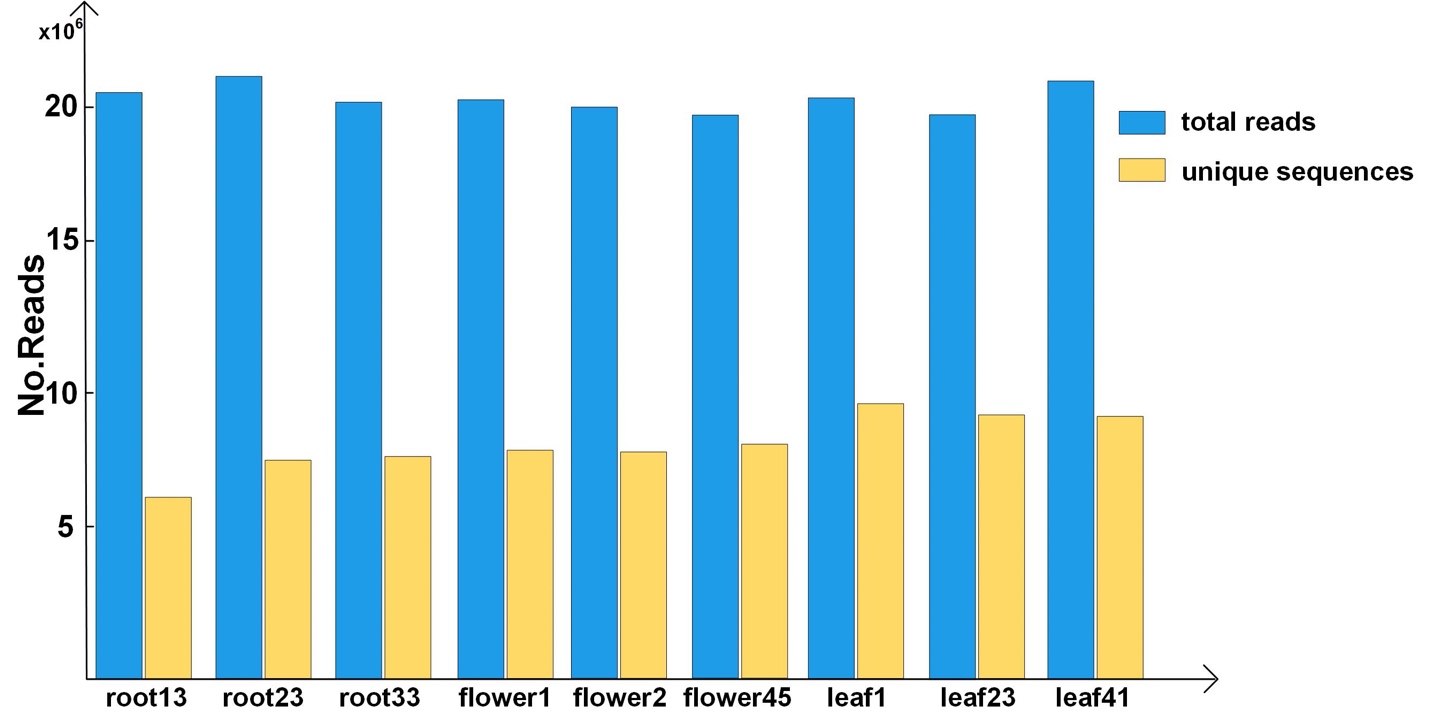
**

**Figure S1.** The number of sequencing reads for each of the nine sRNA-seq libraries. The number of total and unique reads in nine sRNA-seq libraries.


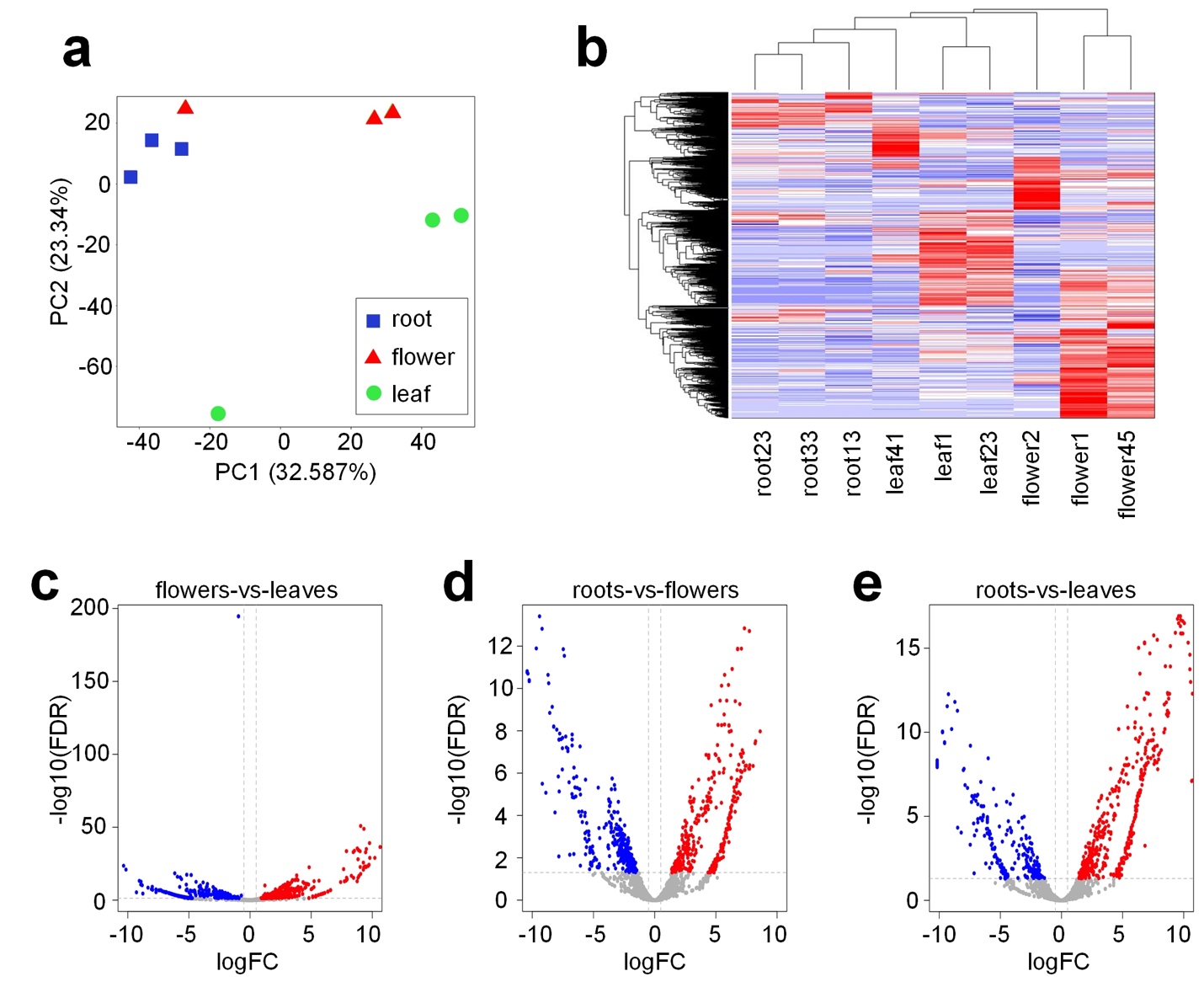


**Figure S2.** PhasiRNAs in *Camellia sinensis* var. *assamica* (YK-10) (**a**) The PCA analysis of phasiRNAs in different tissues. (**b**) The bi-clustering of phasiRNAs in different tissues of *Camellia sinensis* var. *assamica* (YK-10). (**c**) Deregulated phasiRNAs when comparing their expression levels in flowers to those in leaves. Up and downregulated genes in flowers are shown in red and blue dots, respectively. (**d**) Deregulated phasiRNAs when comparing their expression levels in roots to those in flowers. Up and downregulated genes in roots are shown in red and blue dots, respectively. (**e**) Deregulated phasiRNAs when comparing their expression levels in roots to those in leaves. Up and downregulated genes in roots are shown in red and blue dots, respectively.


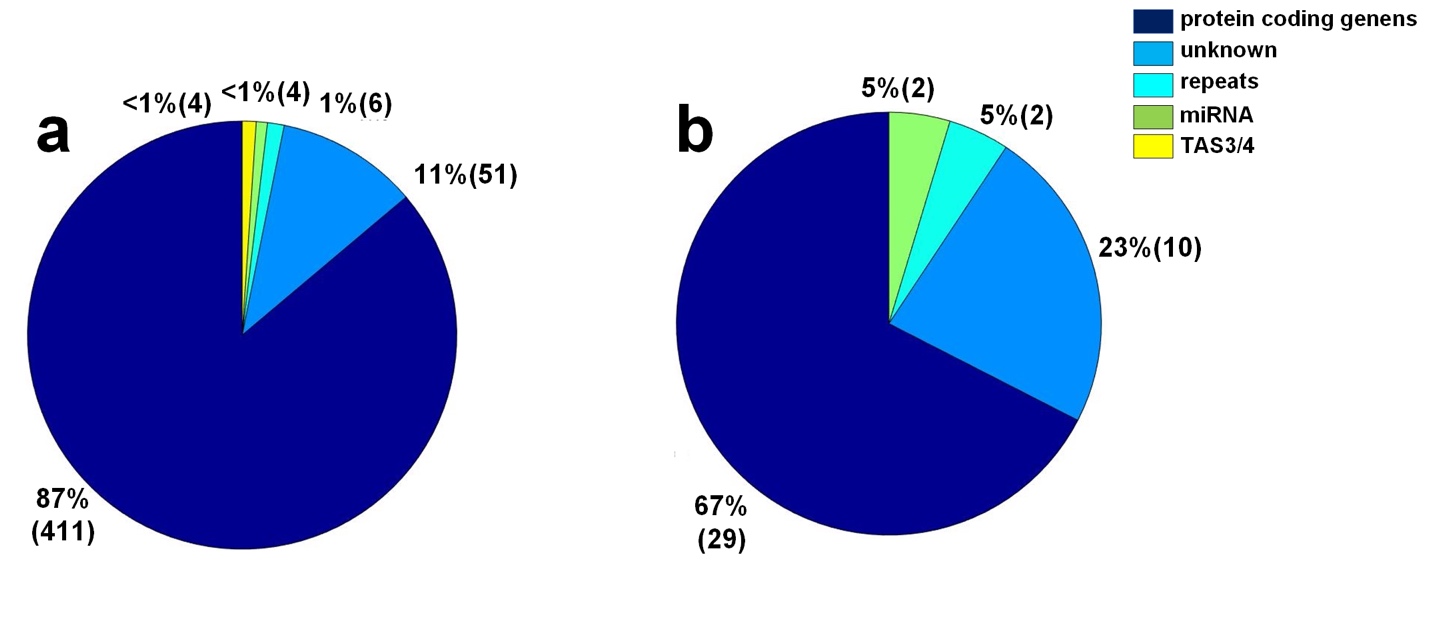


**Figure S3.** The types of molecules of the identified PHAS loci in *Camellia sinensis* var. *assamica* (YK-10). (**a)** The categories of 21 nt PHAS loci. (**b)** The categories of 24 nt PHAS loci.


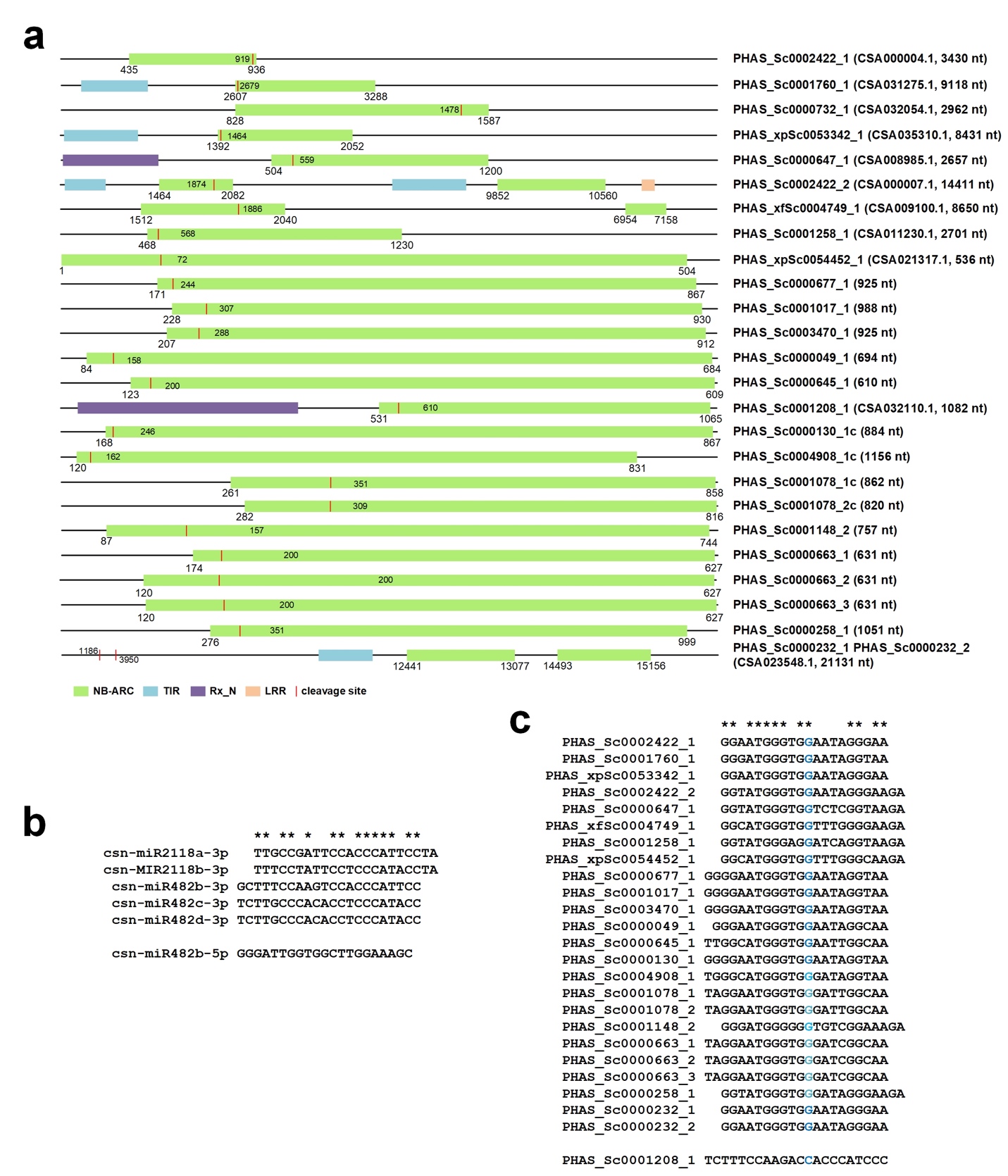


**Figure S4.** The miR482/miR2118 complementary sites on NB-LRR transcripts. **(a)** The distribution of miR482/miR2118 complementary sites on NB-LRR transcripts. The full-length cDNAs of the NB-LRR genes were used to predict the domains if available. The red arrows above the transcripts were the complementary sites of miR482/miR2118 whose cleavage positions, i.e., the blue nucleotide in Part (c), were provided in the parentheses after the miRNA names. The abbreviations of the domains are NB-ARC, Nucleotide-binding domain shared by APAF1, R genes, CED-4; TIR, Toll-like, interleukin-1 receptor resistance protein; Rx\_N, Virus X Resistance Protein N-terminal domain; and LRR, leucine-rich repeat. **(b)** The multiple sequence alignment of miR482 and miR2118 members in tea plant. **(c)** The multiple sequence alignment of miR482 and miR2118 complementary sites on NB-LRR transcripts.


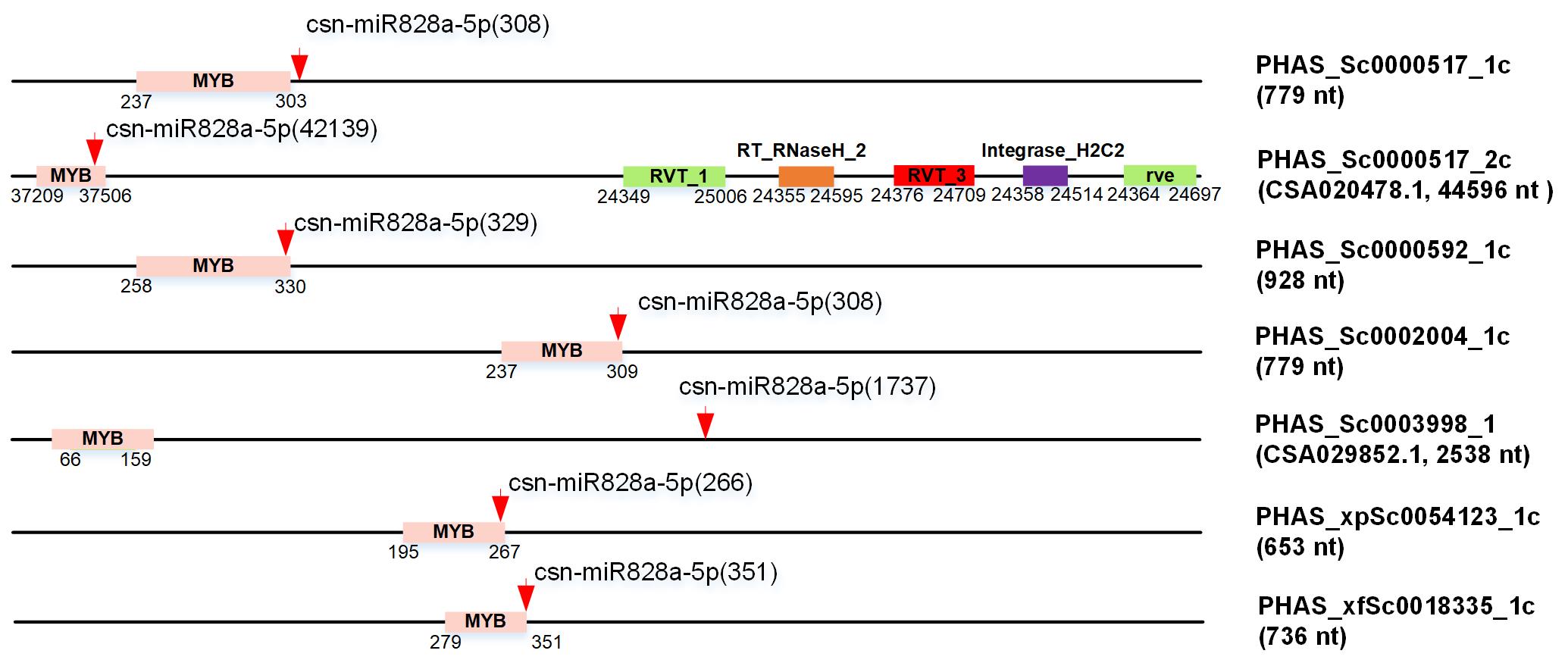


**Figure S5.** The Pfam result of the three identified PHAS loci which are triggered csn-miR828a-5p. The cleavage sites of csn-miR828a-5p at two PHAS loci.

**
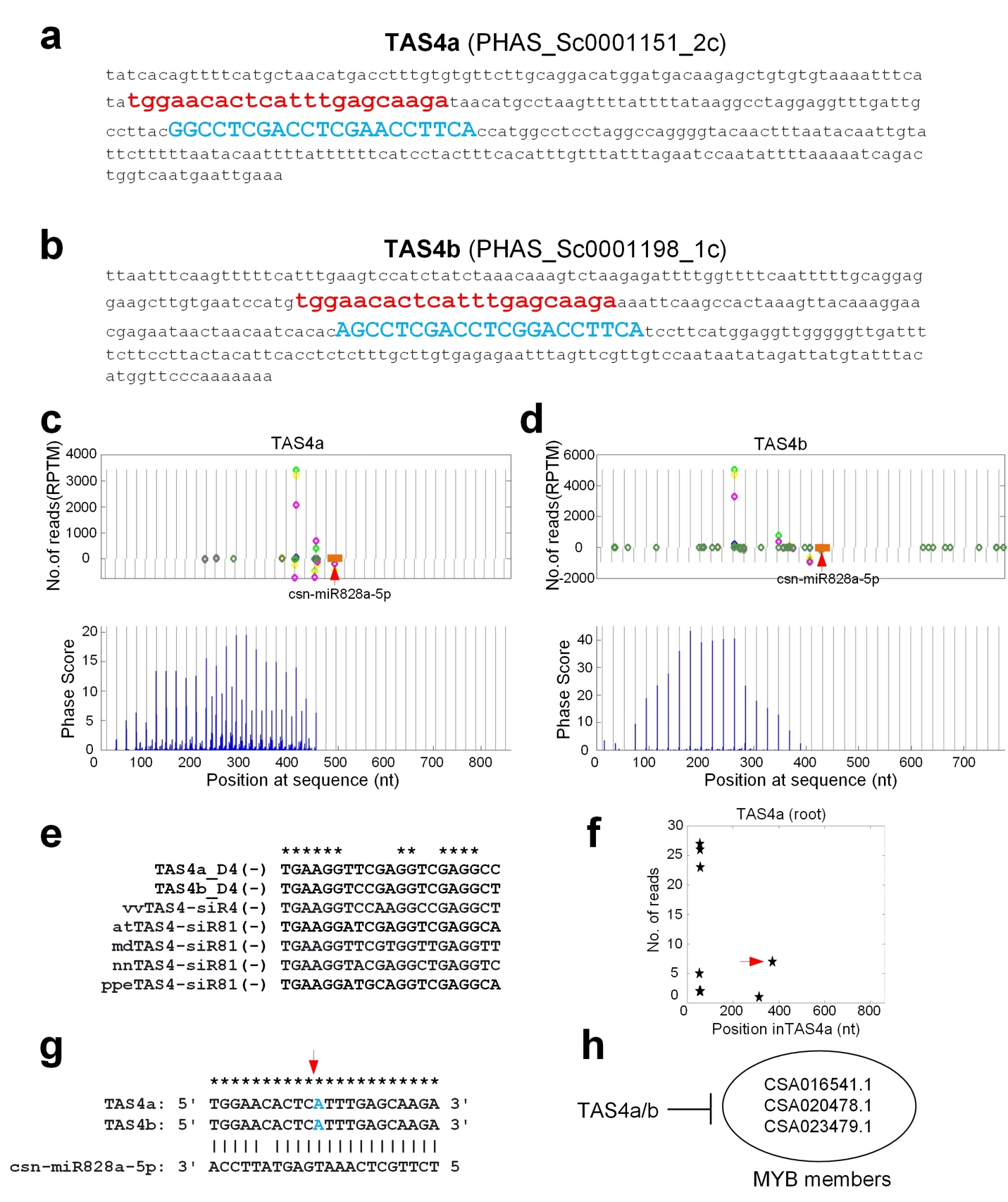
Figure S6.** The TAS4 loci in *Camellia sinensis* var. *assamica* (YK-10). (**a)** The sequence of TAS4a. (**b)** The sequence of TAS4b. In Part (**a**) and (**b**), the red regions are the miR828 complementary sites, and the blue regions are the conserved TAS4 derived tasiRNAs. (**c)** The distribution of small RNA reads in the small RNA profile on TAS4a. **(d)** The distribution of small RNA reads in the small RNA profile on TAS4b. **(e)** The sequence of tasiRNAs in TAS4a and TAS4b. **(f)** The T-plot of TAS4a that is targeted by csn-miR828a-5p. **(g)** The miR828 complementary sites on TAS4a and TAS4b. **(h)** The MYB genes that are targeted by tasiRNAs.


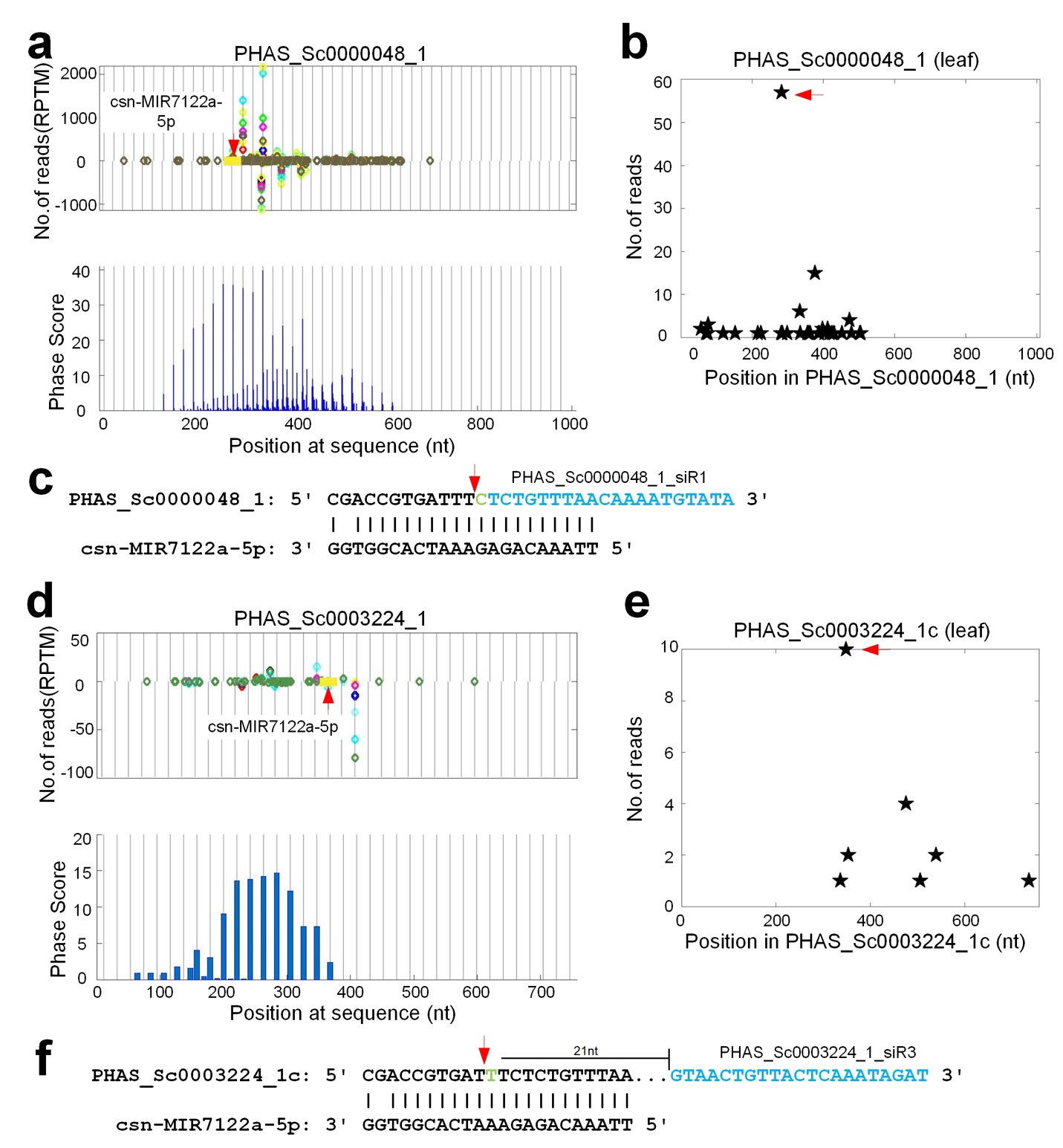


**Figure S7.** Two PHAS loci located at PPR domains are triggered by miR7122. **(a)** The distribution of 21 nt siRNAs and Phase Scores on PHAS_Sc0000048_1. **(b)** - **(c)** The T-plot and complementary site of csn-MIR7122a-5p:PHAS_Sc0000048_1, respectively. **(d)** The distribution of 21 nt siRNAs and Phase Scores on PHAS_Sc0003224_1. **(e)** - **(f)** The T-plot and complementary site of csn-MIR7122a-5p:PHAS_Sc0003224_1, respectively.


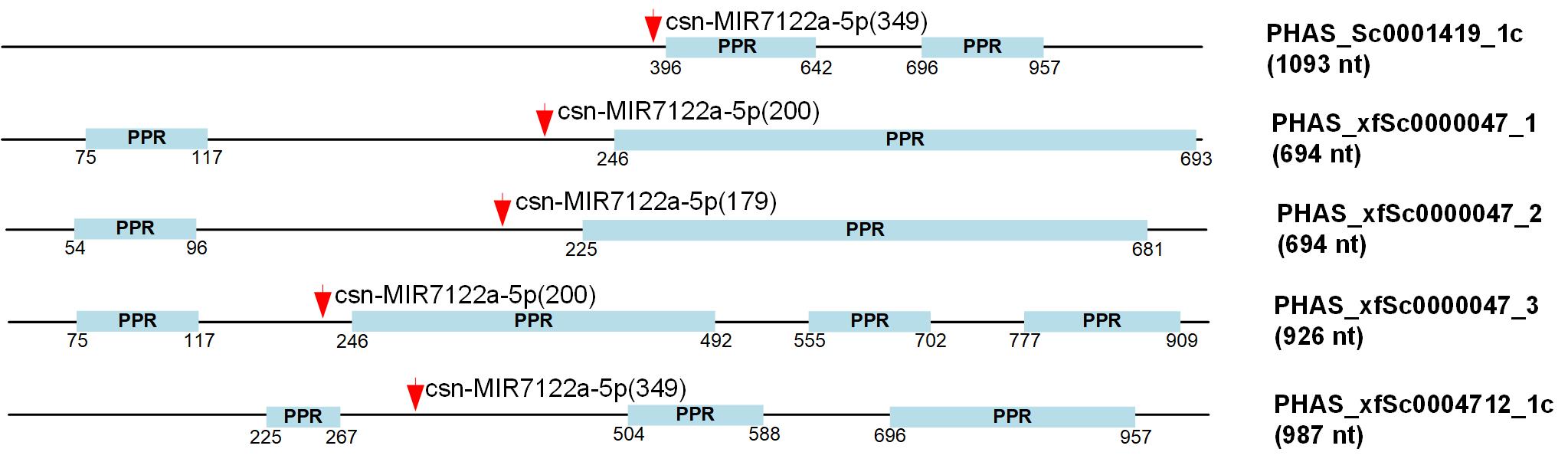


**Figure S8.** The Pfam result of the three identified PHAS loci which are triggered csn-MIR7122a-5p. The cleavage sites of csn-MIR7122a-5p at PHAS loci.


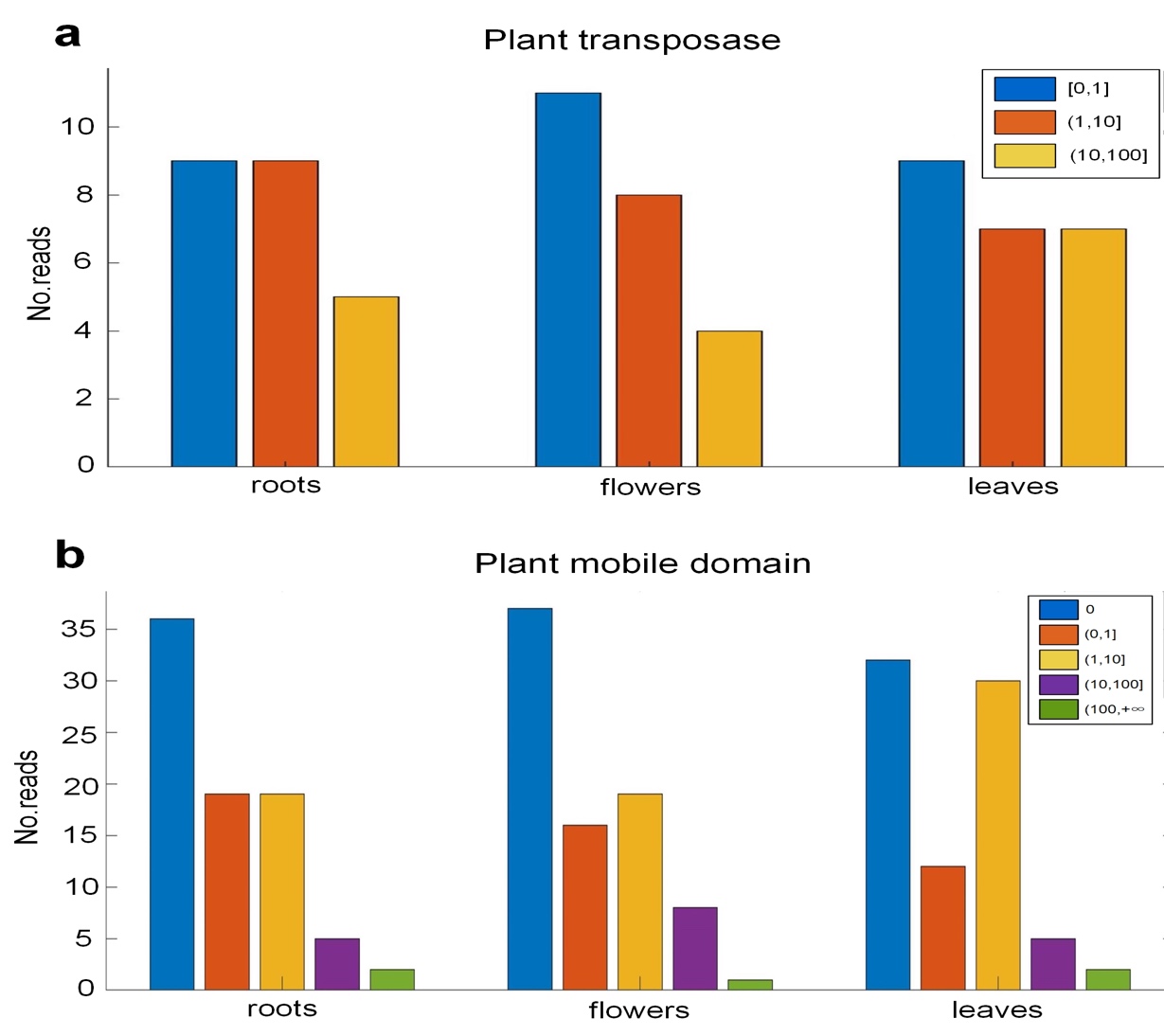


**Figure S9.** The expression level of two gene families. **(a)** The expression level of plant transposase genes in roots, flowers, and leaves. **(b)** The expression level of plant mobile domain genes in roots, flowers, and leaves.


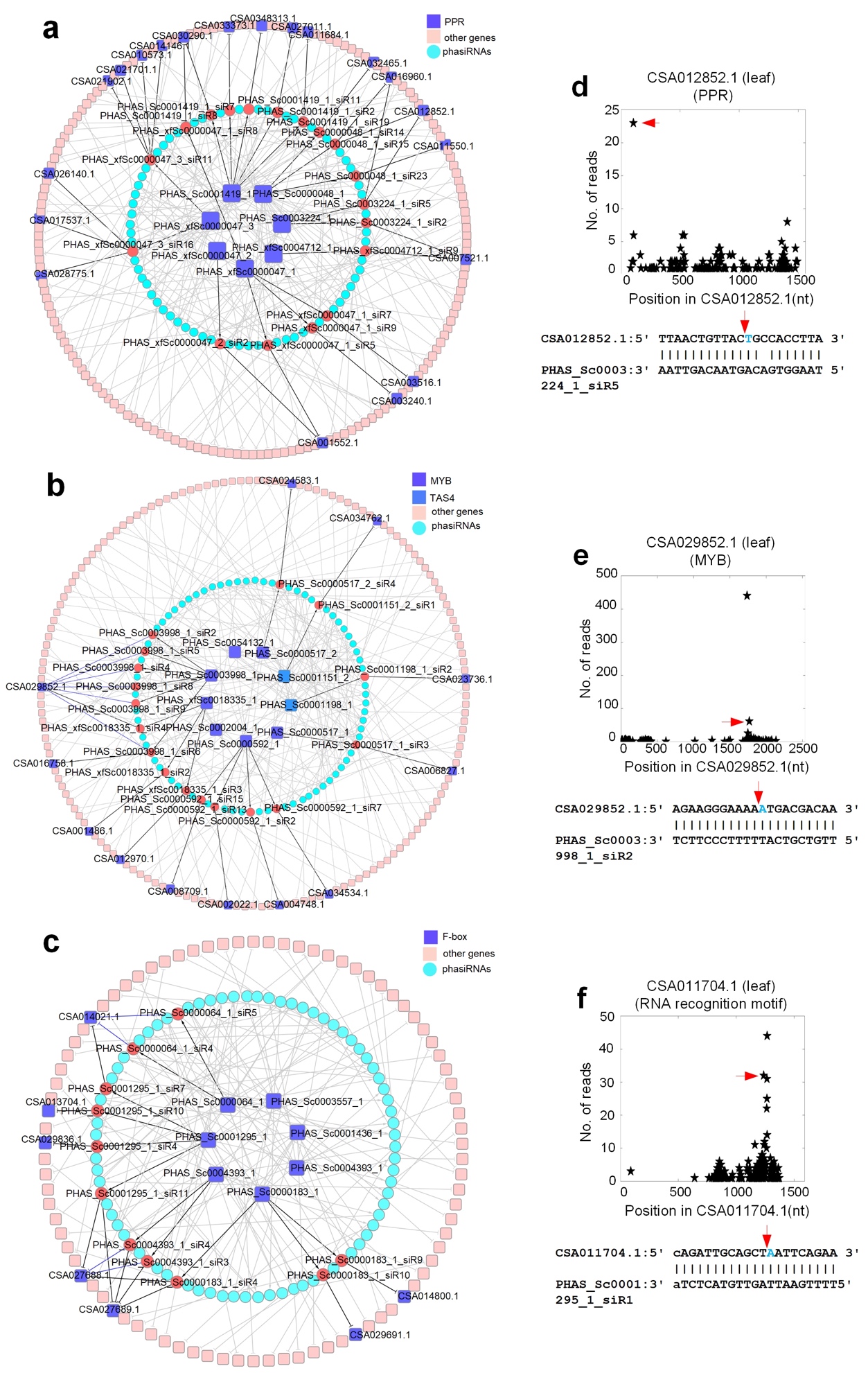


**Figure S10.** The interaction network of genes and phasiRNAs. **(a)** The interaction network of PPR family genes and the phasiRNAs which produced from PPR family genes. **(b)** The interaction network of MYB genes,TAS4 and the phasiRNAs that generated from MYB and TAS4 genes. **(c)** The interaction network of F-box genes and the phasiRNAs which produced from these genes. (**d**) PHAS_Sc0003224_1_siR5: CSA012852.1,a PPR repeat gene. (**e**) PHAS_Sc0003998_1_siR2: CSA029852.1,a MYB gene. (**f**) PHAS_Sc0001295_1_siR1: CSA011704.1. The x-axis is the position on the transcript, and y-axis is the number of reads detected from a position. The arrows in the upper parts correspond to the positions pointed by the arrows of the same colors in the lower parts.


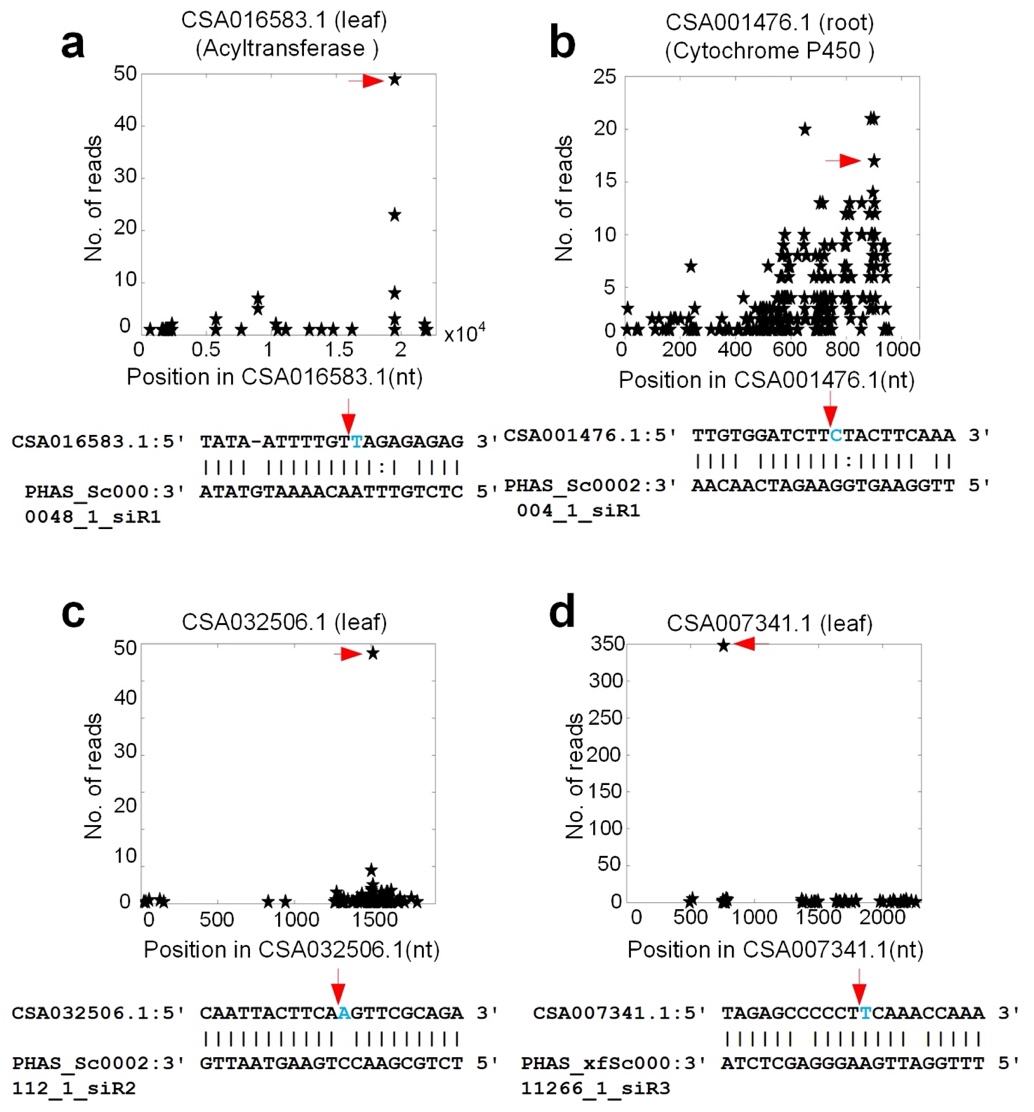


**Figure S11.** The phasiRNA targets in two degradome libraries. The x-axis is the position on the transcript, and y-axis is the number of reads detected from a position. The arrows in the upper parts correspond to the positions pointed by the arrows of the same colors in the lower parts. (**a**) PHAS_Sc0000048_1_siR1:CAS016583.1, an Acyltransferase gene. (**b**) PHAS_Sc0002004_1_siR1:CSA001476.1,a cytochrome P450 gene. (**c**) PHAS_Sc0002112_1_siR2: CSA032506.1. (**d**) PHAS_xfSc00011266_1_siR3: CSA001476.1.


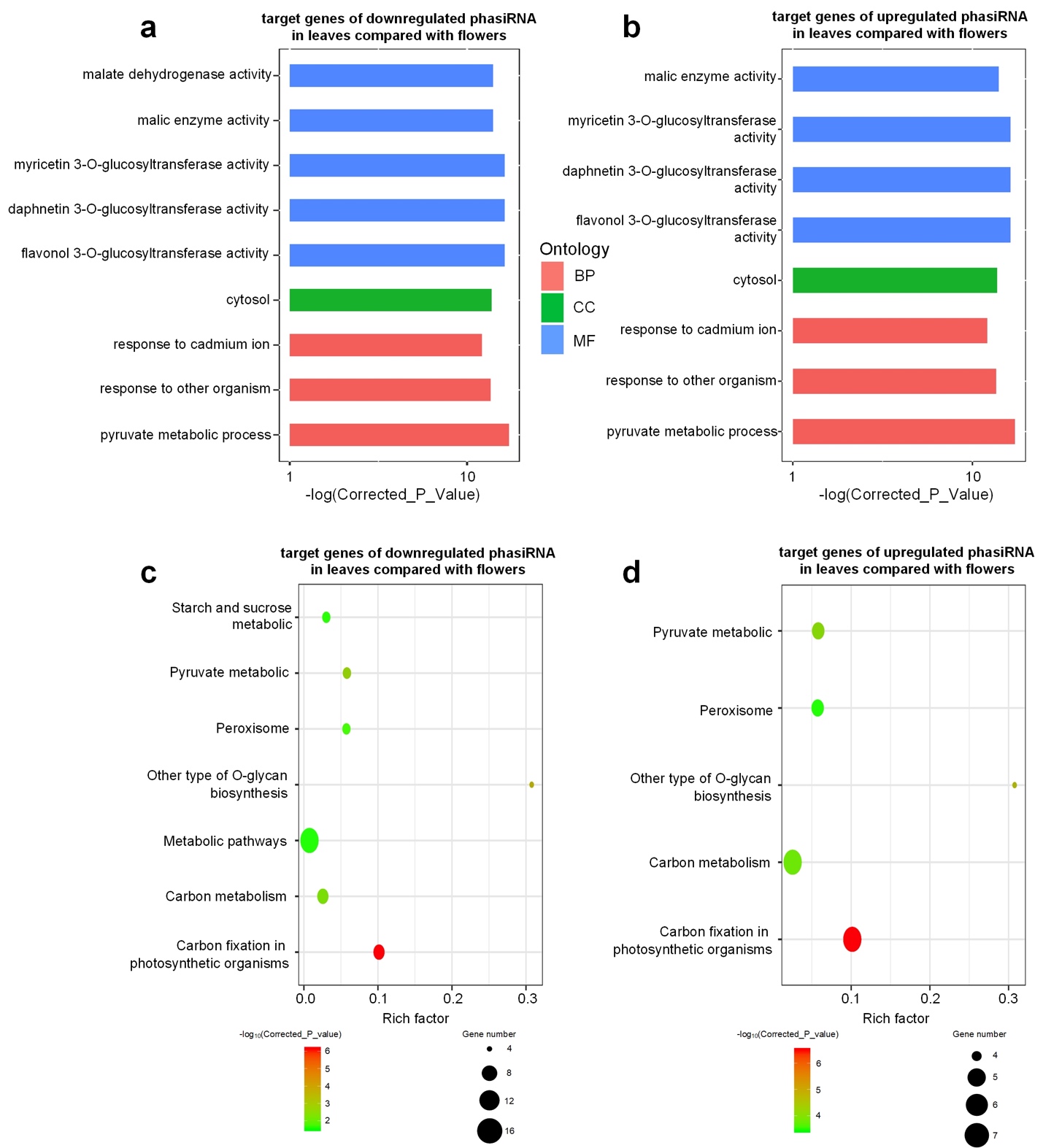
**Figure S12.** Enriched GO terms and KEGG pathways in target genes for deregulated phasiRNAs when comparing RNA-seq profiles of the leaves and flowers samples of *Camellia sinensis* var. *assamica* (YK-10). (a) GO terms of target genes of downregulated phasiRNAs in leaves. (b) GO terms of target genes of upregulated phasiRNAs in leaves. (c) Enriched KEGG pathways of target genes of downregulated phasiRNAs in leaves. (d) Enriched KEGG pathways of target genes of upregulated phasiRNAs in leaves. In Parts (c) and (d), the Rich Factor is calculated by dividing the number of input genes with the KEGG pathway by the total number of genes within the same pathway.


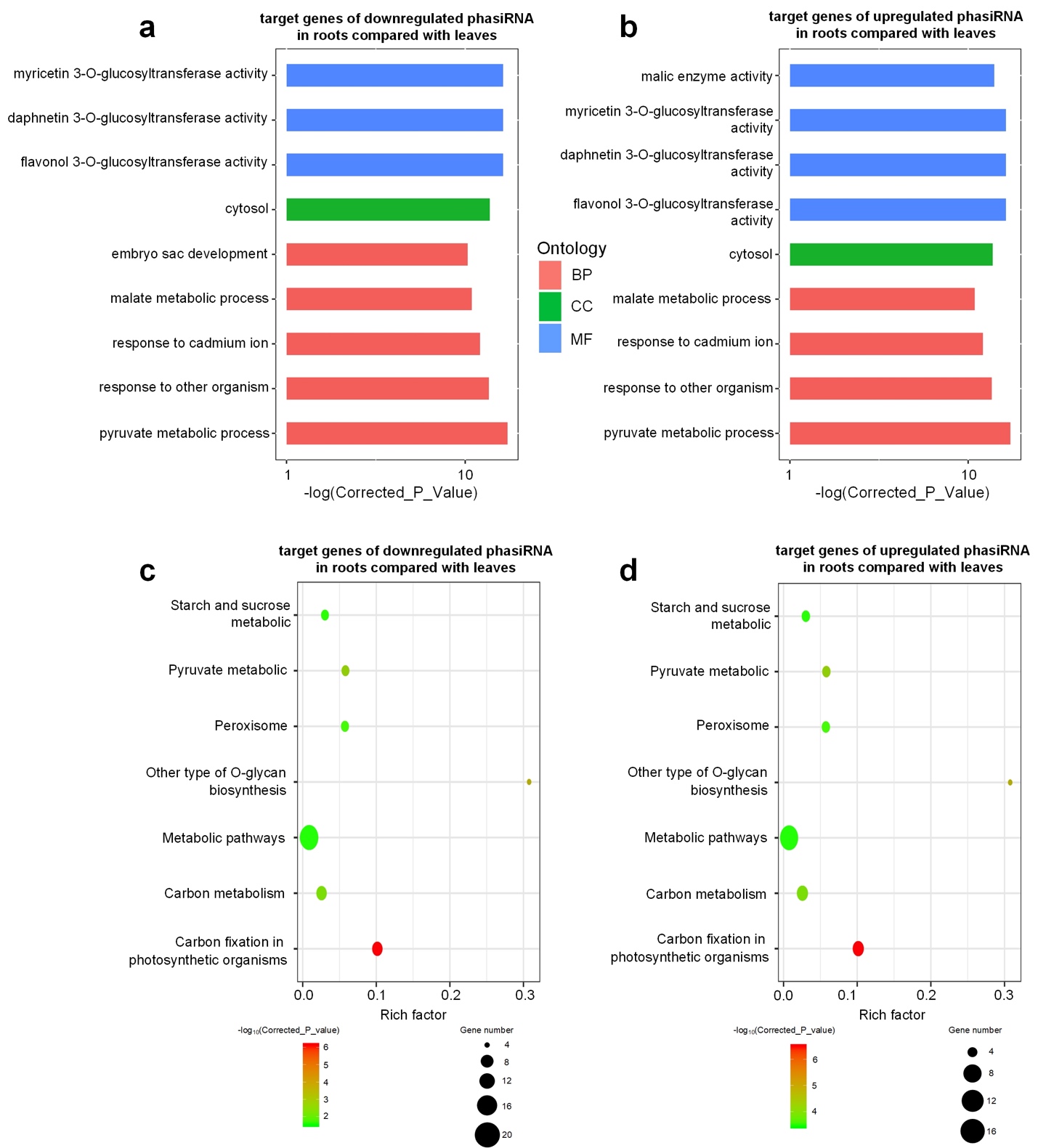
 **Figure S13.** Enriched GO terms and KEGG pathways in target genes for deregulated phasiRNAs when comparing RNA-seq profiles of the roots and leaves samples of *Camellia sinensis* var. *assamica* (YK-10). (a) GO terms of target genes of downregulated phasiRNAs in roots. (b) GO terms of target genes of upregulated phasiRNAs in roots. (c) Enriched KEGG pathways of target genes of downregulated phasiRNAs in roots. (d) Enriched KEGG pathways of target genes of upregulated phasiRNAs in roots. In Parts (c) and (d), the Rich Factor is calculated by dividing the number of input genes with the KEGG pathway by the total number of genes within the same pathway.


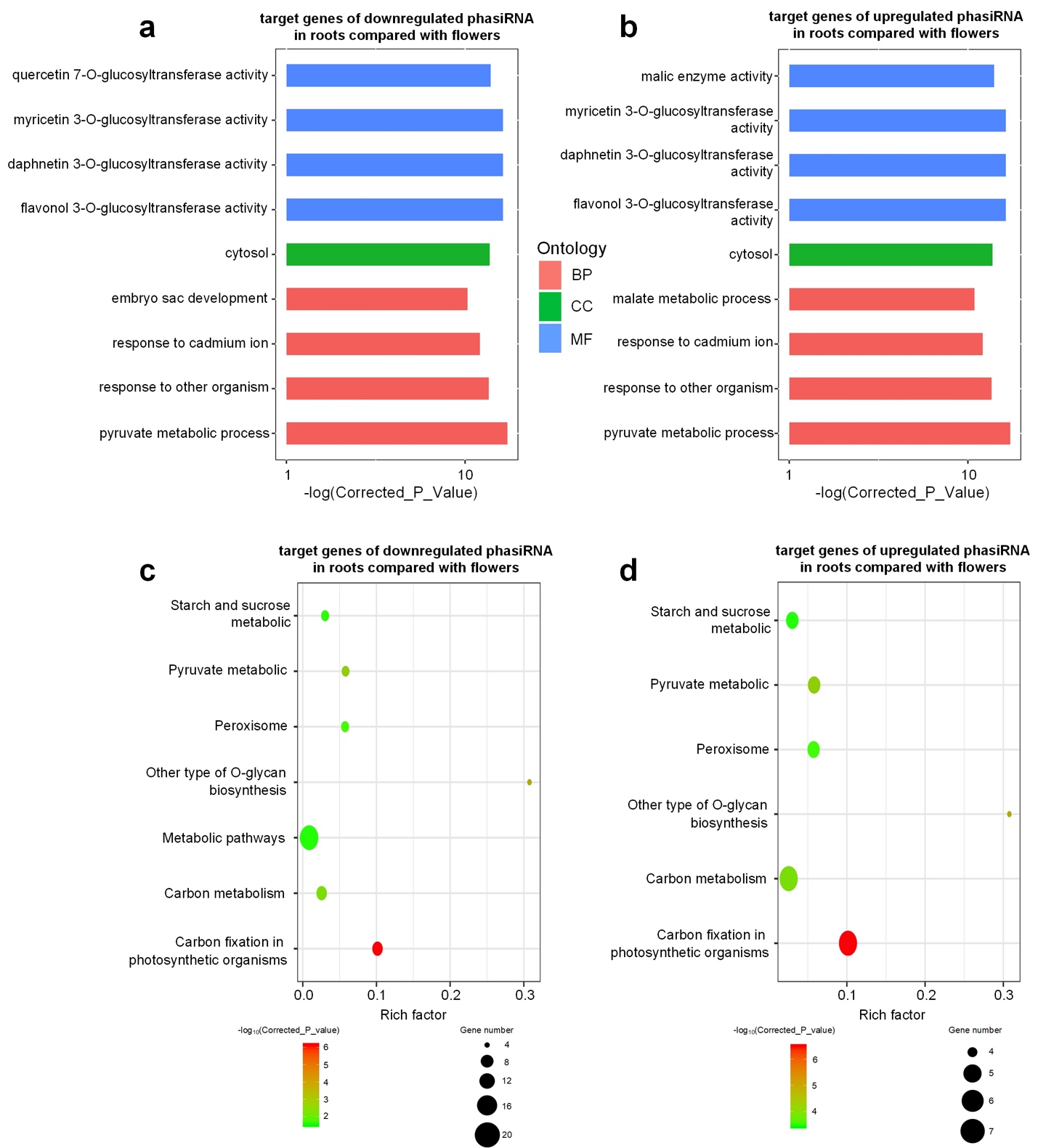
**Figure S14.** Enriched GO terms and KEGG pathways in target genes for deregulated phasiRNAs when comparing RNA-seq profiles of the roots and flowers samples of *Camellia sinensis* var. *assamica* (YK-10). (a) GO terms of target genes of downregulated phasiRNAs in roots. (b) GO terms of target genes of upregulated phasiRNAs in roots. (c) Enriched KEGG pathways of target genes of downregulated phasiRNAs in roots. (d) Enriched KEGG pathways of target genes of upregulated phasiRNAs in roots. In Parts (c) and (d), the Rich Factor is calculated by dividing the number of input genes with the KEGG pathway by the total number of genes within the same pathway.
